# Integrating metagenomic binning with flux balance analysis to unravel syntrophies in anaerobic CO_2_ methanation

**DOI:** 10.1186/s40168-022-01311-1

**Published:** 2022-08-03

**Authors:** Nicola De Bernardini, Arianna Basile, Guido Zampieri, Adam Kovalovszki, Beatrix De Diego Diaz, Elisabetta Offer, Nantharat Wongfaed, Irini Angelidaki, Panagiotis G. Kougias, Stefano Campanaro, Laura Treu

**Affiliations:** 1 Department of Biology, University of Padova, Via U. Bassi 58/b, 35121 Padua, Italy; 2 Department of Environmental Engineering, Technical University of Denmark, 2800 Kgs. Lyngby, Denmark; 3 Department of Chemistry, University of Navarra, Pamplona, Spain; 4 Department of Biotechnology, Faculty of Technology, Khon Kaen University, Khon Kaen, 40002 Thailand; 5 Department of Chemical and Biochemical Engineering, Technical University of Denmark, Kgs, DK-2800 Lyngby, Denmark; 6 Hellenic Agricultural Organization DEMETER, Soil and Water Resources Institute, Thermi, Thessaloniki, Greece; 7 CRIBI Biotechnology Center, University of Padova, 35131 Padova, Italy

**Keywords:** Metagenomics, Flux balance, Metabolic modelling, Anaerobic CO_2_ methanation, Biogas upgrading

## Abstract

**Background:**

Carbon fixation through biological methanation has emerged as a promising technology to produce renewable energy in the context of the circular economy. The anaerobic digestion microbiome is the fundamental biological system operating biogas upgrading and is paramount in power-to-gas conversion. Carbon dioxide (CO_2_) methanation is frequently performed by microbiota attached to solid supports generating biofilms. Despite the apparent simplicity of the microbial community involved in biogas upgrading, the dynamics behind most of the interspecies interaction remain obscure. To understand the role of the microbial species in CO_2_ fixation, the biofilm generated during the biogas upgrading process has been selected as a case study. The present work investigates via genome-centric metagenomics, based on a hybrid Nanopore-Illumina approach the biofilm developed on the diffusion devices of four ex situ biogas upgrading reactors. Moreover, genome-guided metabolic reconstruction and flux balance analysis were used to propose a biological role for the dominant microbes.

**Results:**

The combined microbiome was composed of 59 species, with five being dominant (> 70% of total abundance); the metagenome-assembled genomes representing these species were refined to reach a high level of completeness. Genome-guided metabolic analysis appointed *Firmicutes* sp. GSMM966 as the main responsible for biofilm formation. Additionally, species interactions were investigated considering their co-occurrence in 134 samples, and in terms of metabolic exchanges through flux balance simulation in a simplified medium. Some of the most abundant species (e.g., *Limnochordia* sp. GSMM975) were widespread (~ 67% of tested experiments), while others (e.g., *Methanothermobacter wolfeii* GSMM957) had a scattered distribution. Genome-scale metabolic models of the microbial community were built with boundary conditions taken from the biochemical data and showed the presence of a flexible interaction network mainly based on hydrogen and carbon dioxide uptake and formate exchange.

**Conclusions:**

Our work investigated the interplay between five dominant species within the biofilm and showed their importance in a large spectrum of anaerobic biogas reactor samples. Flux balance analysis provided a deeper insight into the potential syntrophic interaction between species, especially *Limnochordia* sp. GSMM975 and *Methanothermobacter wolfeii* GSMM957. Finally, it suggested species interactions to be based on formate and amino acids exchanges.

Video Abstract

**Supplementary Information:**

The online version contains supplementary material available at 10.1186/s40168-022-01311-1.

## Introduction

Anaerobic digestion (AD) is a fundamental step in the carbon cycle that converts complex organic matter into simpler molecules under anoxic conditions. This process is naturally present in many different ecological niches (e.g., marine sediments, terrestrial marshlands, digestive system of eukaryotes), while being purposefully utilized by biotechnological approaches. For one, industrial AD generates biogas, a renewable alternative to natural gas composed of a mixture of methane (CH_4_) (50–75%) and carbon dioxide (CO_2_) (25–50%), with minor amounts of contaminants [1]. Since the concentration of CH_4_ obtained from biogas reactors rarely exceeds 60%, in recent years more research effort has been put into discovering new approaches to biologically couple CO_2_ contained in biogas with hydrogen (H_2_). In such a way, the CH_4_ content of the biogas is purified to almost 100%, and at the same time higher productivity rates are achieved [2]. This concept, commonly termed biogas upgrading or power-to-gas, is therefore a viable CO_2_ valorization method and allows for the injection of the resulting biomethane directly into the natural gas grid [1].

An important factor for the establishment of a stable AD microbiome is biofilm formation. Interactions of microorganisms often rely on close proximity, thus spatial structure of microbial communities is a key factor for cooperation and can drive community dynamics [3]. Biofilms are complex structures constituted by prokaryotic cells glued together by extracellular polymeric substances (EPS) [4] secreted to create a protected growth environment under unfavorable scenarios [5, 6]. The EPS confers mechanical stability to the biofilm, and promotes bacterial adhesion to surfaces.

The structure of microbial communities is also influenced by cross-feeding of electron donors, auxotrophy, and the energetic gain/burden resulting from the degradation/synthesis of essential nutrients (e.g., vitamins, cofactors and amino acids) of different microorganisms. These aspects are particularly important since many metabolic activities occurring in anaerobic digesters take place at their thermodynamic limits; thus, maintaining an optimal tradeoff between operational parameters is fundamental for the methanation efficiency. For example, the more H_2_ and CO_2_ partial pressure rises, the more methanogenesis becomes exergonic and the Gibbs free energy change generated from the degradation of amino acids and volatile fatty acids increases. Therefore, methanogens play an important role to keep H_2_ concentrations sufficiently low, allowing metabolic pathways inhibited by high H_2_ concentrations involved in organic matter degradation to take place [5].

For biogas upgrading to work, microbial syntrophism is essential [7]. The core of the process is indeed the syntrophy between hydrogenotrophic methanogenic archaea and acetate-consuming bacteria and the bidirectional conversion of acetate, CO_2_ and H_2_ to CH_4_, to optimize partial pressures. Studies on syntrophies are insightful in elucidating the metabolic potential of individual microorganisms, but also provide knowledge that can be transferred to whole microbiomes. As shown in the past, syntrophic acetate oxidizing bacteria (SAOB) can convert acetate into CO_2_ and H_2_ [8]. More specifically, SAO is functionally the reverse of an acetogenic pathway (reductive acetyl-coenzyme A), also known as Wood-Ljungdahl pathway (WLP) [9]. The overall scheme of the WLP is conserved in both bacterial and archaeal metabolism: they share the same carbonyl branch, but differ in the methylic branch, in terms of their enzymes, electron carriers and cofactors. Additionally, when using the WLP for energy generation and carbon fixation, acetogens produce acetate as an end product, whereas hydrogenotrophic archaea produce CH_4_ [10]. A potential alternative pathway for acetate oxidation was identified in a *Thermotoga* and *Clostridium drakei* species, consisting of an incomplete WLP coupled with the glycine cleavage system [11, 12].

The relevant role of biogas-producing microorganisms is yet to be fully unveiled, especially when it comes to understanding trophic relations among the microbes. Despite the huge advancements of recent years, the biological mechanism behind biogas production is still regarded as a “black box”. In the case of AD, a deeper knowledge of the microbial ecological relationships could help elucidate the whole process and could eventually provide biotechnological ways to circumvent problems leading to reactor fluctuations and failures, or to improve bioaugmentation strategies [13–15].

To investigate such a system, random sequencing allows de novo identification of uncultured microorganisms, reliable annotation and gene identification. Furthermore, newer and promising techniques, such as “third-generation sequencing”, have demonstrated to improve the assembly quality and completeness values of the recovered genomes, thus, enriching the knowledge about diverse microbiomes, including AD [16–18]. The generation of high-quality metagenome-assembled genomes (MAGs) is a key step in building genome scale metabolic models (GSMMs) for flux balance analysis (FBA). However, only recently the development of reliable automated reconstruction methods allowed the use of MAGs to build single-species and community models in a fast and scalable manner [19, 20]. The application of these methods has allowed the inspection of complex and biologically mediated biogas production systems [21].

In light of the above considerations, this study aims to unveil the trophic relationships and microbial roles behind biofilm establishment in four different thermophilic up-flow reactors for ex-situ biogas upgrading. In depth analysis of specific key genes that take part in biofilm system formation was performed. Furthermore, computational analyses were adopted to reconstruct the metabolic maps of the key microbial species taking part in the process. Finally, flux balance analysis was applied to simulate the behavior of the community and for a deeper investigation of metabolic exchanges behind the establishment of the biofilm community.

## Material and methods

### Reactors’ set-up and characteristics at steady state

Operation and set-up of the reactors fed with exhausted digestate from Snertinge biogas plant (Denmark) is described in detail in previous study [22]. Reactors operated under thermophilic conditions (55 °C) and were continuously provided with gas composed of H_2_, CH_4_ and CO_2_ (with ratio 62:23:15) through different diffusion devices. For reactors 1 to 4, these devices were composed of three stainless steel diffusers with pore size of 0.5 μm, 0.2 μm, and an Al_2_O_3_ ceramic membrane with pore size of 0.4 μm and 1.2 μm, respectively. These four reactors were operated for six different periods in which gas retention time or gas recirculation rate was modified. Biofilms were sampled at steady state of period 6 [22].

### DNA extraction and metagenome sequencing

Biofilm was formed on the diffusers of each reactor in variable amounts (2–14 g) and approximately 1 g per each sample was used for DNA extraction. The extraction was carried out using PowerSoil® DNA isolation Kit (MO BIO laboratories, Carlsbad, CA, USA) following manufacturer instructions and adding an initial step of purification (P:C:IAA). Quality and quantity of each of the extractions was assessed using gel electrophoresis, NanoDrop 2000 (ThermoFisher Scientific, Walthman, MA, USA) and Qubit fluorometer (Life Technologies, Carlsbad, CA, USA).

A sequencing strategy combining short and long-reads was adopted as previously described [18] and was based on NextSeq 500 platform (Illumina Inc., San Diego, CA) and Oxford Nanopore MinION single-molecule sequencers. For the Illumina sequencing, library preparation was performed using Nextera DNA Flex Library Prep Kit (Illumina Inc., San Diego CA), while for the Oxford Nanopore MinION, the SQK rapid sequencing kit was selected (Oxford Nanopore Technologies, Oxford, UK). Libraries were sequenced with Illumina with paired-end and with FLO-MIN106 R9 flow cell on a MinION device (Oxford Nanopore Technologies, Oxford, UK) at the CRIBI biotechnology center sequencing facility (University of Padova, Italy).

### Genome-centric metagenomics and statistics

Guppy v2.3.7 + e041753 basecaller was adopted to translate raw electrical signal to nucleotide sequence for the long-read sequencing. Trimmomatic (v0.39) was used for Illumina read filtration. High-quality reads were independently assembled with three software, namely Spades (v3.14.0-0), OPERA-MS, and Unicycler (v0.4.8-beta). QUAST_1 (v.4.1) was used to calculate assembly statistics. Scaffolds shorter than 1 kb were removed. Bowtie 2 (v2.2.4), in combination with checkM (v1.0.3), enabled the determination of the coverage of each scaffold, by aligning the reads of each sample back onto the assembly. Metagenomic binning was performed with a mixed approach involving Metabat2 (v2.12.1) and MaxBin2 (v2.2.6). CheckM (v1.0.3) was used to determine basic metrics (completeness, contamination, relative abundance values) of the final MAGs. Taxonomic assignments were performed with GTDB-Tk (v1.3.0) and verified by using the 16S rRNA gene sequences aligned on SILVA 138 as previously suggested [23].

The phylogenetic tree was obtained using PhyloPhlAn 3.0 (3.0.51) [24] and drawn using iTOL [25]. Protein-encoding genes were predicted using Prodigal (v2.6.2) with default parameters and associated with KEGG IDs using EggNOG (v2.0.1-1) and Diamond (v0.9.22.123). KEGG IDs were associated with the corresponding KEGG modules to determine their completeness values by using an in-house developed pipeline [26]. Additional gene finding and annotation was performed on the five dominant MAGs by Rapid Annotation using Subsystem Technology (RAST) to order genes into subsystems, subcategories and categories, according to the SEED classification by sequence attribution to protein families (FIGfams) [27]. To obtain the most complete possible annotation regarding genes responsible for exopolysaccharide biosynthesis, a targeted investigation was performed using Hidden Markov Models (HMM) search implemented in KEMET [26]. The software was used with the option --fixed_ko_list and the dedicated ko list (Supplementary Dataset S1) was manually defined starting from SEED classifications.

Profile abundance of all MAGs present in the biofilm were obtained considering the AD metagenomes present in the MiGa Biogas microbiome database [28]. The raw reads have been aligned to the 59 MAGs to obtain the coverage profile. CheckM (v.1.0.3) was used to compute the relative abundance of each species in each experiment. Based on literature information the results were summarized in 67 identifiers by averaging the MAGs abundances across the replicates of each metagenome (Supplementary Dataset S2). Eventually, only experiments containing at least 1% of the five dominant species present in the biofilm have been retained in the barplot. SparCC was used to compute Pearson correlation coefficient between the coverage profiles of the 59 MAGs [29].

A literature-guided metabolic reconstruction was manually performed based on genes and pathways present in the five dominant MAGs retrieved from the integration of EggNOG (v 2.0.1-1) and Diamond (v0.9.22.123) annotations (Supplementary Dataset S3). A visual representation of the most relevant metabolic pathways was created combining the KEGG module completeness information and the absence/presence of key enzymatic activities. For the operon identification in MAGs, the Operon Mapper software [30] was used, setting a threshold of > 0.8 probability to be in the same operon.

### Probe design and fluorescence in situ hybridization

Clustal Omega [31] was used to align 16S rRNA gene sequences of taxonomic-related species to obtain genome-specific fluorescence in situ hybridization (FISH) probes of the two dominant microbes. The more divergent regions were selected in order to maximize the mismatches on non-target organisms and probe positions were 345-368 and 1121-1146 for *Methanothermobacter wolfeii* GSMM957 and *Limnochordia* sp. GSMM975, respectively. *In silico* assessment of probe hybridization efficiency and risk of potential non-target matches was performed using MathFISH software [32]. Samples collected from the four reactors were inoculated and maintained in anaerobic flasks with Basal Anaerobic medium (BA) using CO_2_ as carbon source [18]. Samples were collected from the growing cultures and hybridized on 4% (wt/vol) paraformaldehyde-fixed cultures as previously described by *et al.* (2001) [33]. The specific probe targeting *Limnochordia* sp. GSMM975 (5′- TTT AGA GTC CCC AAC TTA ATG CTG G -3′) was marked with 6-carboxyfluorescein, 6-FAM (green). *M. wolfeii* GSMM957 genome-specific FISH probe (5′-TTG CGT GCA TTG CGG AGG TTT C-3′) was marked with Alexa 647 (red).

The samples were pretreated with lysozyme (5 mg/mL) and proteinase K (1 μg/mL). Furthermore, after fixation, the cell permeability was increased through five freeze/thaw cycles (− 20 to 55 °C) [34, 35]. Hybridized probes were investigated by confocal scanning laser microscopy (Leitz DM RBE microscope; true confocal system TCS SP with a multiband confocal imaging spectrophotometer [Leica Microsystems, Heidelberg, Germany]) equipped with He and Ar/Ne lasers (set at approximately 38% of maximum intensity). The laser wavelengths used for the detection of 6- FAM and Alexa 647 are 488 and 650 nm, respectively. Leica Application Suite X (LAS X, LASX) was used to capture the images and for the rendering.

### Metabolic modelling and flux balance analysis

The MAGs assigned to the most abundant species (relative abundance higher than 5%) were selected for in silico metabolic modelling. Their genome-scale metabolic models (GSMMs) were created with gapseq v.1.1 (commit: 6ae3a6a) [20] starting from the corresponding quality-filtered MAGs. The only exception is *Acetomicrobium* sp. GSMM972, which was substituted with a closely related MAG named AS02xzSISU_38 from the MiGa Biogas microbiome database [28], due to its low completeness level (checkM 77.97%) [36]. The genomic identity between these two MAGs exceeded 95% (average nucleotide identity: 98.53%), indicating that they belong to the same species with a high confidence [37].

During the GSMM reconstruction of each microbe, the most appropriate metabolic reaction universe (bacterial or archaeal) was adopted, along with the default gene matching parameters to limit the gene/pathway search according to taxonomic ranges. Draft GSMMs were generated specifying the biomass reaction (gram positive, gram negative, or archaeal) when enough taxonomic information was available, and were automatically inferred otherwise. Default parameters were used for the bit score thresholds (i.e. -l and -u) that define reactions with or without sequence evidence. During the gap-filling step, a minimum bit score of 100 was considered to include a reaction among the core ones, and the experimental conditions (e.g., high H2 concentration) were taken into account to adjust reaction direction: (Supplementary Dataset S4). Quality of GSMMs was assessed using MEMOTE (v.0.13.0). Finally, using Micom (v.0.10.1), the species-specific GSMMs were manipulated to construct a single community model, which simultaneously accounts for the exchange fluxes between individuals, and individuals with the environment [38]. Analysis of the metabolic exchanges among the different microbes was performed by setting the microbial community biomass accumulation as objective and utilizing the cooperative tradeoff approach. This method allows to get the highest unique solution for each species growth rate whilst enforcing a sup-optimal community growth rate set a priori as a fraction of the maximum hypothetical growth rate. The relative abundances of the different species were used to account for the community composition during the simulations. Cplex (v.12.8.0.0) was adopted as the optimization solver in all the model reconstructions and analyses [39].

To simulate the microbial growth in the reactors, an in silico basic anaerobic (BA) medium was defined based on its measured composition (Supplementary Dataset S5). Such medium was used as a proxy for the nutrient-depleted digestate composition, thereby reproducing the lack of carbon sources, while providing essential trace elements. The concentration of single ions and metabolites was calculated from the BA composition protocol, and was converted into units of mmol/L [40]. These values were then scaled throughout the entire medium based on their relative concentration. A maximum uptake rate of 1000 mmol/gDW/h was allowed only for water, while more constrained bounds between 1 and 100 mmol/gDW/h were imposed on all the other metabolites. The uptake rate boundary values were expressed in units of mmol/L for in silico growth medium fluxes, and in units of mmol/gDW/hr for the internal fluxes [41]. Additional bounds regarding the consumption of H_2_ and CO_2_ and the production of CH_4_ were fixed in the model, based on the experimental measurements of the reactors [22]. For given experimental conditions, these bounds would limit the solution space to be explored by the solver within realistic ranges for H_2_ and CO_2_ uptake and for CH_4_ production. Specifically, the measured input gas flow rate (QIN) [L/LRd] was converted to liters of gas injected per day, and then to mmol/gDW/hr using the molar volume of an ideal gas at 298 K (24.46 L/mol) and an estimated dry mass per liter of the microbial community equal to 1.17 gDW/L. The concentration of the microbial community used to rescale the fluxes was defined based on the measure of volatile suspended solid obtained growing a simplified hydrogenotrophic community in BA medium [42]. The uptake rate (i.e., lower and upper bound) of CO_2_ and H_2_ were fixed at a 1:4 ratio, specifically at 1.20 and 5.11 mmol/gDW/h, while the minimum export rate of CH_4_ was fixed at 1.15 mmol/gDW/h.

Cytoscape (v.3.8.2) was used for the graphical representation of the predicted exchanges between microbes. The depicted fluxes were divided between amino acids and metabolites directly involved in methanogenesis (CO_2_, H_2_, CH_4_, acetate, pyruvate, and formate). Only fluxes larger than 0.3 mmol/gDW/h have been plotted. The sensitivity of exchanges selected to target interventions on medium composition or genus abundances was quantified using the elasticity coefficient, computed as it is implemented in Micom. This parameter was used to quantify how strongly the univariate changes (e.g., intervention on parameters) in the system impact a given exchange flux [38]. The fraction of maximal community growth rate enforced in all the analysis involving a simulation is 0.5.

## Results

### Taxonomic and functional composition of the microbiome

**Fig. 1:**
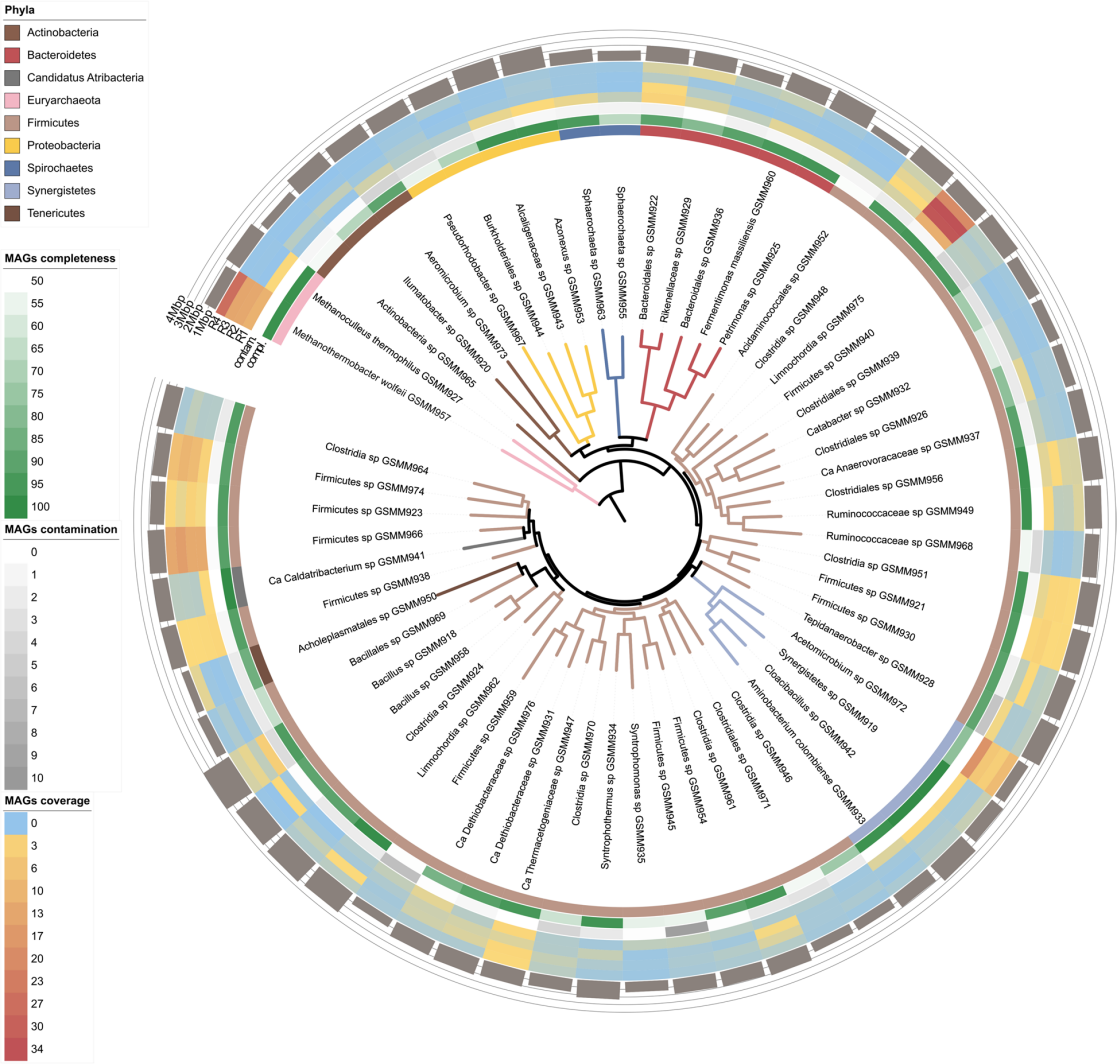
Phylogenetic tree of the biofilm microbial species. Phylogenetic tree obtained for the MAGs extracted from the four samples. From outside inwards: MAG size in Mbp (barplot), heatmap with the MAG relative abundance in the four reactors, contamination/completeness values and taxonomic assignment. The phylogenetic tree was obtained with PhyloPhlAn 3.0 and rerooted on the archaeal MAGs; colors were assigned according to the taxonomic assignment at phylum level

The gas injection systems of the four biogas upgrading reactors (R1–R4), on top of which biofilm formed, were composed of stainless-steel diffusers in R1 and R2 (pore size of 0.5 μm and 0.2 μm) and consisted of Al_2_O_3_ ceramic membranes in R3 e R4 (0.4 μm and 1.2 μm). The biofilm community formed in the reactors proved to be very simple given that only 59 MAGs in total were recovered during this study (Fig. 1). 60% of the MAGs were of high-quality, more than 90% complete and less than 5% contaminated, according to the minimum information about metagenome-assembled genome guidelines (MIMAG) [43]. The 59 MAGs were taxonomically assigned to eight different phyla (Fig. 1A). Most of the microbiota were represented by five species, namely *Limnochordia* sp. GSMM975, *M. wolfeii* GSMM957, *Acetomicrobium* sp. GSMM972, *Firmicutes* spp. GSMM966, and GSMM974, accounting for almost 70% of the overall community in each reactor: (Supplementary Dataset S6). The process of MAG reconstruction was of high quality as demonstrated by the almost completely closed genome obtained for the two dominant microbes, i.e., *Limnochordia* sp. GSMM975 and *M. wolfeii* GSMM957 (Supplementary material). All biofilms populations presented the same taxa, with *Limnochordia* sp. GSMM975 being typically the dominant species (on average 25% of the whole community). The only exception was the community developed in reactor R1 (Fig. 1B), where *Acetomicrobium* sp. GSMM972 was the most abundant species covering nearly 20% of the community. Moreover, *M. wolfeii* GSMM957 was the dominant member of the community in R4 having nearly 26% relative abundance.

**Fig. 2:**
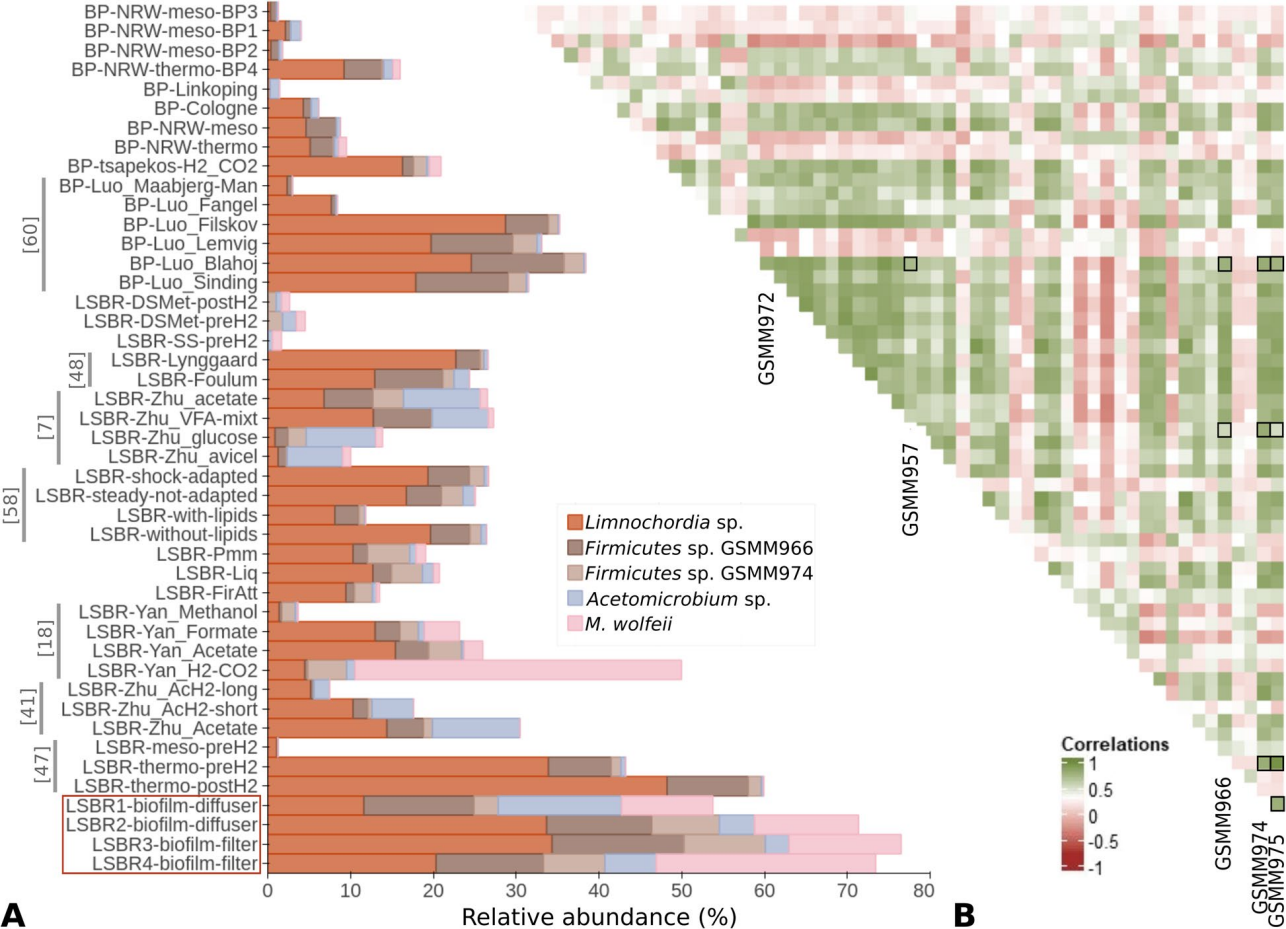
Heatmap of the Pearson correlation coefficients calculated from the MAGs coverage values and histogram of the main species abundance. **A** Histogram of the relative abundance for the most abundant species in the biofilm observed in AD metagenomes available in the MiGa Biogas microbiome database. References for the corresponding works are reported on the left side, and the samples of the present work are marked in red. **B** Pearson correlation coefficient calculated considering the relative abundance profile of the MAGs present in the biofilm community in a global dataset based on the AD metagenomes available in MiGa Biogas microbiome database. The positive correlations are reported in green color, the negative correlations in red color and the correlations for which the *p* value was lower than the threshold (< 0.05) in grey color. The marked squares highlight the positive correlations between the most abundant species in the biofilm

Putative obligate and/or facultative syntrophies among members of the microbiome were investigated by co-occurrence analysis, also considering the similarity in relative abundance across the present and previous experiments (Fig. 2). The evaluation included a dataset of 134 samples collected from batch and full-scale anaerobic digestion systems [36]. This analysis demonstrates that within the biofilm community several MAGs reciprocally co-occur at a statistically significant level (Fig. 2B). The five dominant MAGs were significantly and positively correlated between them (*r* > 0.5). Specifically, *Limnochordia* sp. GSMM975 and *Firmicutes* sp. GSMM966 shared the highest correlation (0.96), followed by *Acetomicrobium* sp. GSMM972 and *M. wolfeii* GSMM957 (0.71). All other pairs between the five species (including *Firmicutes* sp. GSMM974) show relatively lower correlation values, which may suggest their ability to establish facultative mutualistic associations.

The relevance of the dominant microbes was demonstrated by the correlation between their abundance profiles not only in the biofilm, but generally in the AD microbiome. In fact, analysis of the presence of *Limnochordia* sp. GSMM975 and the other four species in the AD database confirmed that it is widespread in AD systems (Fig. 2A). In particular, *Limnochordia* sp. was found among the dominant MAGs (relative abundance > 1%) in the 55% of full and laboratory scale biogas reactors operating at thermophilic condition [36].

### Molecular mechanisms involved in biofilm formation

**Fig. 3:**
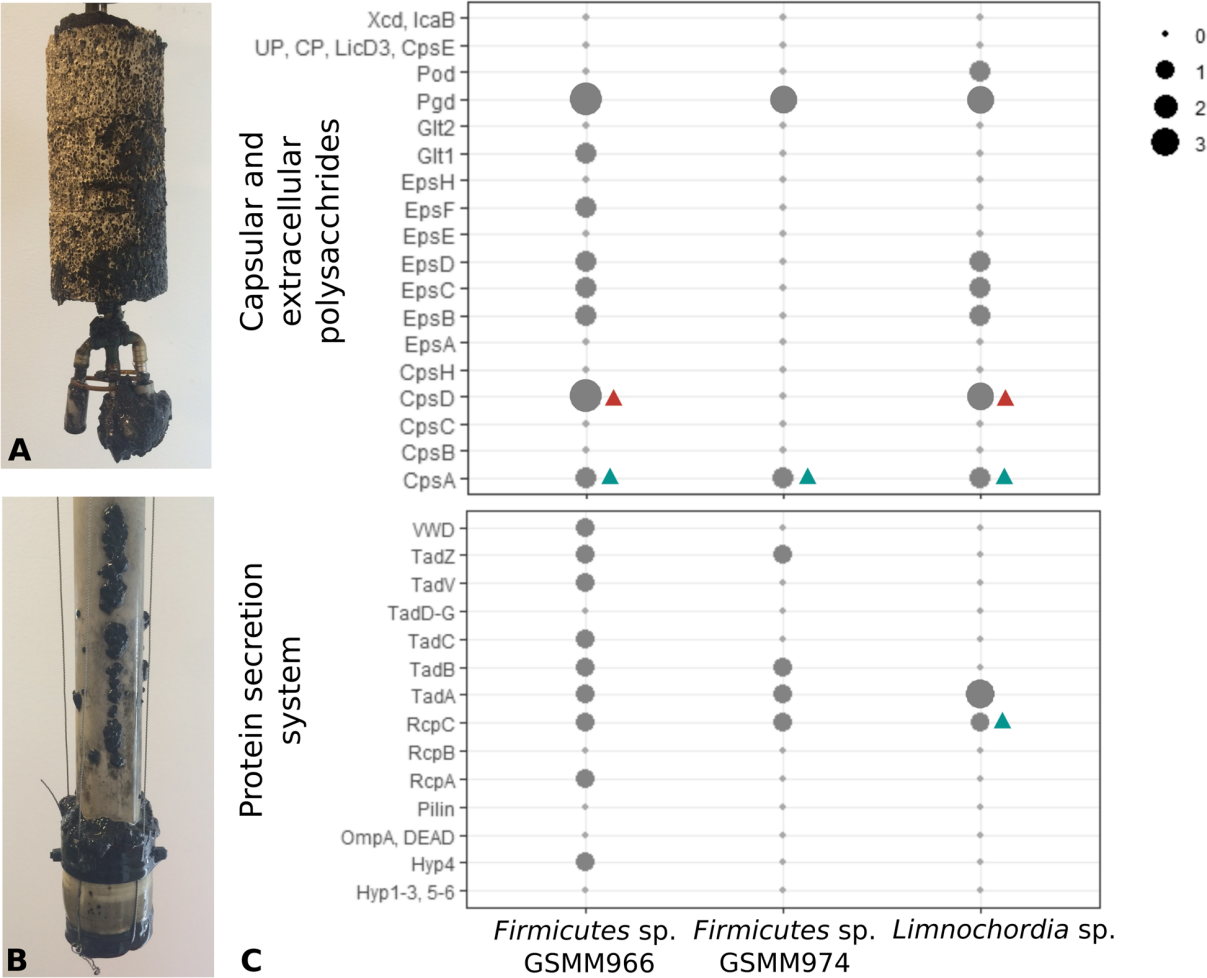
Biofilm accumulated on the membrane and diffuser of the reactors and the detected biofilm-related genes. **A**, **B** Picture of biofilm samples growing on the diffusers used to inject gas in the reactors: the stainless-steel diffusers above (**A**) and the ceramic membrane below (**B**). **C** Sets of genes belonging to modules associated with biofilm development detected in the genome of the abundant species. Dots sizes shows the number of copies found per genome. The green triangles refer to a result retrieved by EggNOG annotation alone, while the red triangles refer to a result obtained through HMM search

The pivotal role of the dominant species in AD is further evidenced in the biofilm matrix formation. EPS secretion by microorganisms generates an embedding material crucial for the development of a protected growth environment [44], such as the one accumulated on the membrane and diffuser of the upgrading reactors of the current experiment (Fig. 3A). Therefore, the analysis of the contribution of each species to EPS synthesis is important to shed light on potential mechanisms that can be exploited to manipulate microbial communities and influence AD systems.

Results showed that *Firmicutes* spp. GSMM966, GSMM974 and *Limnochordia* sp. GSMM975 have a predominant role in biofilm formation. Specifically, these MAGs possess two sets of genes organized in operons, which are involved in the generation of the EPS layer with different but complementary roles (Fig. 3B). The first operon includes: a manganese-dependent protein-tyrosine phosphatase (EpsB, EC 3.1.3.48), a tyrosine-protein kinase transmembrane modulator (EpsC), a tyrosine-protein kinase (EpsD, EC 2.7.10.2) and, only in GSMM966, two glycosyltransferases (EpsF EC 2.4.1.- and Glt1), belonging to an EPS-related gene family [45]. The second operon, which is related to extracellular polysaccharides, includes a set of polysaccharide deacetylases (Pgd) and it was found in the three genomes with some variations (Fig. 3B). A third set of genes found in the three MAGs is related to capsular polysaccharide synthesis and sugar transfer (cpsA, cpsD). Additionally, among the predicted operons (Supplementary Dataset S7), *Firmicutes* sp. GSMM966 has one with genes classified as protein secretion system (type II/IV pili synthesis), including pilus assembly genes flp, cpaB, cpaC, cpaE, cpaF, tadB, and tadC that may be partially relevant in the process. The same operon identified in *Firmicutes* sp. GSMM974 includes only three genes organized in a functional unit, with the exception of cpaC. Moreover, analysis of the KEGG modules revealed that the pathway “polyamine biosynthesis, arginine => agmatine => putrescine => spermidine” (M00133), potentially relevant in biofilm stabilization, is complete in all the five dominant MAGs present in the microbial community.

### Genome-guided metabolic reconstruction

**Fig. 4:**
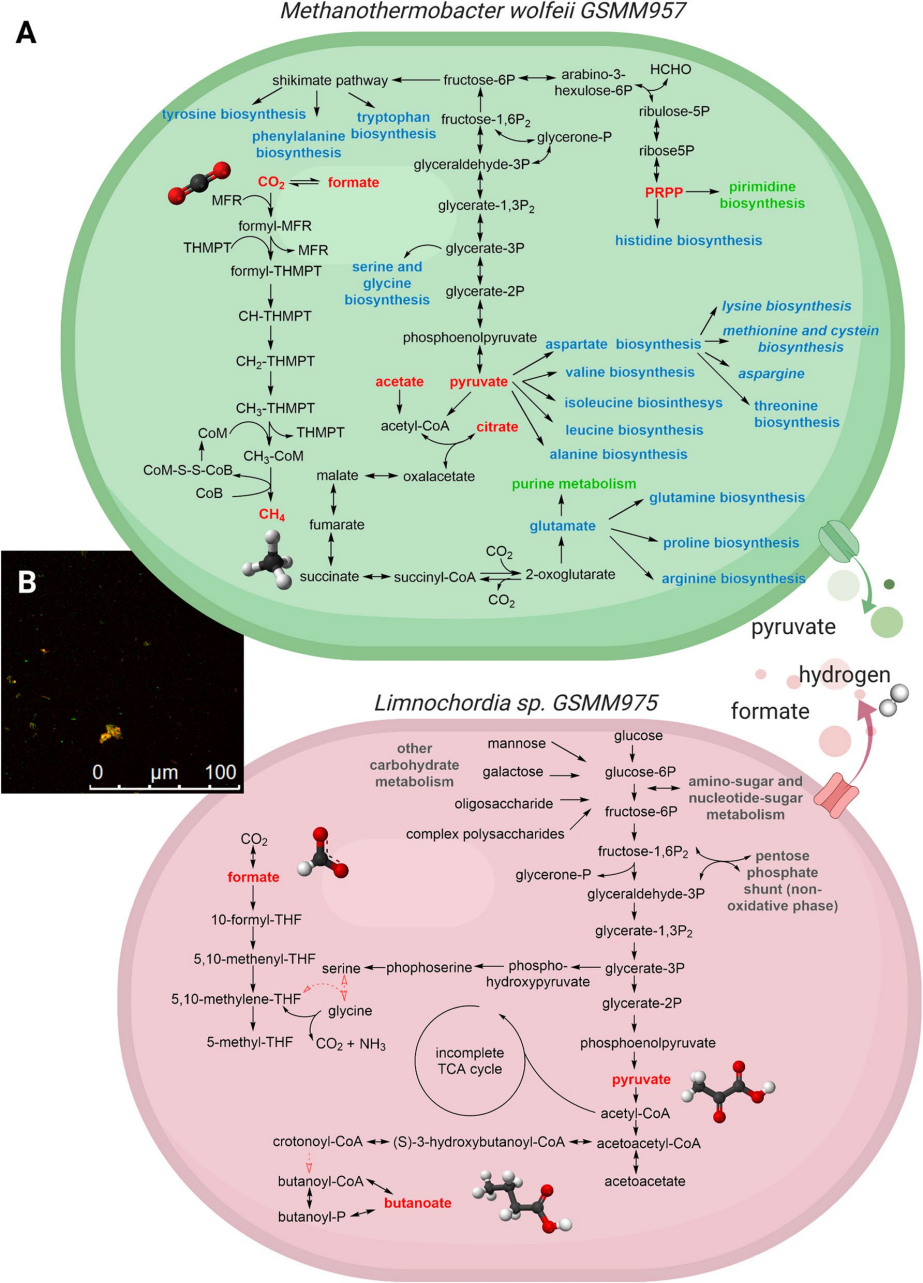
**A** Scheme of metabolic maps obtained for the two dominant species. Metabolic map summarizing the main metabolic pathways detected in *M. wolfeii* GSMM957 and *Limnochordia* sp. GSMM975. Putative metabolites exchanged between the species are also reported. **B** FISH image of the microbial community. *M. wolfeii* GSMM957 is labeled with red probes and *Limnochordia* sp. GSMM975 with green probes

Metabolic pathway reconstruction and biological role interpretation of the 59 MAGs was performed investigating the completeness level of 394 KEGG modules (Supplementary Dataset S3). Manual inspection indicated that 12% are widespread “core” modules, while most modules presented a scattered distribution in terms of presence/absence in the biofilm community. Analysis of module completeness underlined that both archaeal species (*Methanoculleus thermophilus* GSMM927 and *M. wolfeii* GSMM957) have complete hydrogenotrophic-specific modules, that are “F420 biosynthesis” (M00378) and “methanogenesis, CO_2_ => methane” (M00567). In particular, the metabolic map of the two dominant microbes, *M. wolfeii* GSMM957 and *Limnochordia* sp. GSMM975, revealed very complex metabolisms (Fig. 4). Being hydrogenotrophic, the archaeon can perform methanogenesis from H_2_ and CO_2_, but also from formate, and it has complete pathways for the biosynthesis of coenzymes M and F420. Apart from these essential cofactors, it can synthesize a molybdenum-based coenzyme for formylmethanofuran dehydrogenase (fwdA, fmdA, or K00200), thiamine, which is important for the metabolism of amino acids, and siroheme, which is a sulfite reductase cofactor. On the other hand, the metabolic map of *Limnochordia* sp. GSMM975 has a complex central carbon metabolism, including not only a complete glycolysis pathway and an almost complete pentose-phosphate shunt, but also the ability to use fructose, mannose, and galactose as additional carbon sources. Concerning gluconeogenesis, *M. wolfeii* GSMM957 can produce metabolites up to fructose-6-phosphate and synthesize phosphoribosyl pyrophosphate (PRPP). PRPP is pivotal for the metabolism of purines, as well as for tetrahydromethanopterin (H4MPT) biosynthesis, which is fundamental for the conversion of CO_2_ to CH_4_ [46, 47]. Both microbial species have the pathway for terpenoid biosynthesis. The archaeon, however, produces isopentenyl pyrophosphate (IPP) and dimethylallyl pyrophosphate (DMAPP) using mevalonate as intermediate. Contrariwise, the biosynthetic pathway of the bacterium is mevalonate-free. Due to its ability to fix molecular nitrogen, the amino acid biosynthetic pathways of *M. wolfeii* GSMM957 were investigated in more detail. It appeared to be able to synthesize almost the entire set of amino acids, since its pathways of valine, isoleucine, leucine, tryptophan, glutamate, glutamine, aspartate, asparagine, arginine, and lysine biosynthesis are complete. While some gaps were found in the biosynthetic pathways of the two sulfuric amino acids, there is only one enzymatic step missing for tyrosine and phenylalanine biosynthesis and no gaps in the shikimate-chorismate pathway.

Vice versa, the amino acid metabolism of *Limnochordia* sp. GSMM975 has a low synthetic potential, suggesting the presence of numerous auxotrophies. Moreover, *Limnochordia* sp. GSMM975 does not have a complete WLP, since it lacks the last enzymes in the methyl branch. It also does not have access to the catalytic activity to form acetate from the methyl moiety. Therefore, it cannot perform SAO by simply reversing the WLP, as *T. phaeum* can [48]. However, other ways to sustain the CO_2_ reduction pathway and its fixation into biomass components were scrutinized. Specifically, the reductive glycine pathway (RGP) and the glycine synthase-reductase pathway (GSRP), both utilizing the Glycine Cleavage System (GCS), were tentatively reconstructed. The original CDSs of the three GCS proteins, encoded by four functional identifiers, were detected in the genome: their loci were at small genomic distance, suggesting an operon-like organization (COG1003, COG0403, COG0509, COG0404). Other two genes encoding proteins of the RGP were identified in the genome of *Limnochordia* sp. GSMM975, namely the glycine/serine hydroxymethyltransferase (glyA or SHMT) and serine dehydratase (sdaA).

For a deeper investigation of the hypothesized syntropy, specific primers for *Limnochordia* sp. GSMM975 and *M. wolfeii* GSMM957 were generated, and their potential interaction was inspected through FISH (Fig. 4B; Supplementary material). The results from probes hybridization clearly highlight the co-localization of the two species within the same flocs.

### In silico simulation of the core biofilm community

**Fig. 5:**
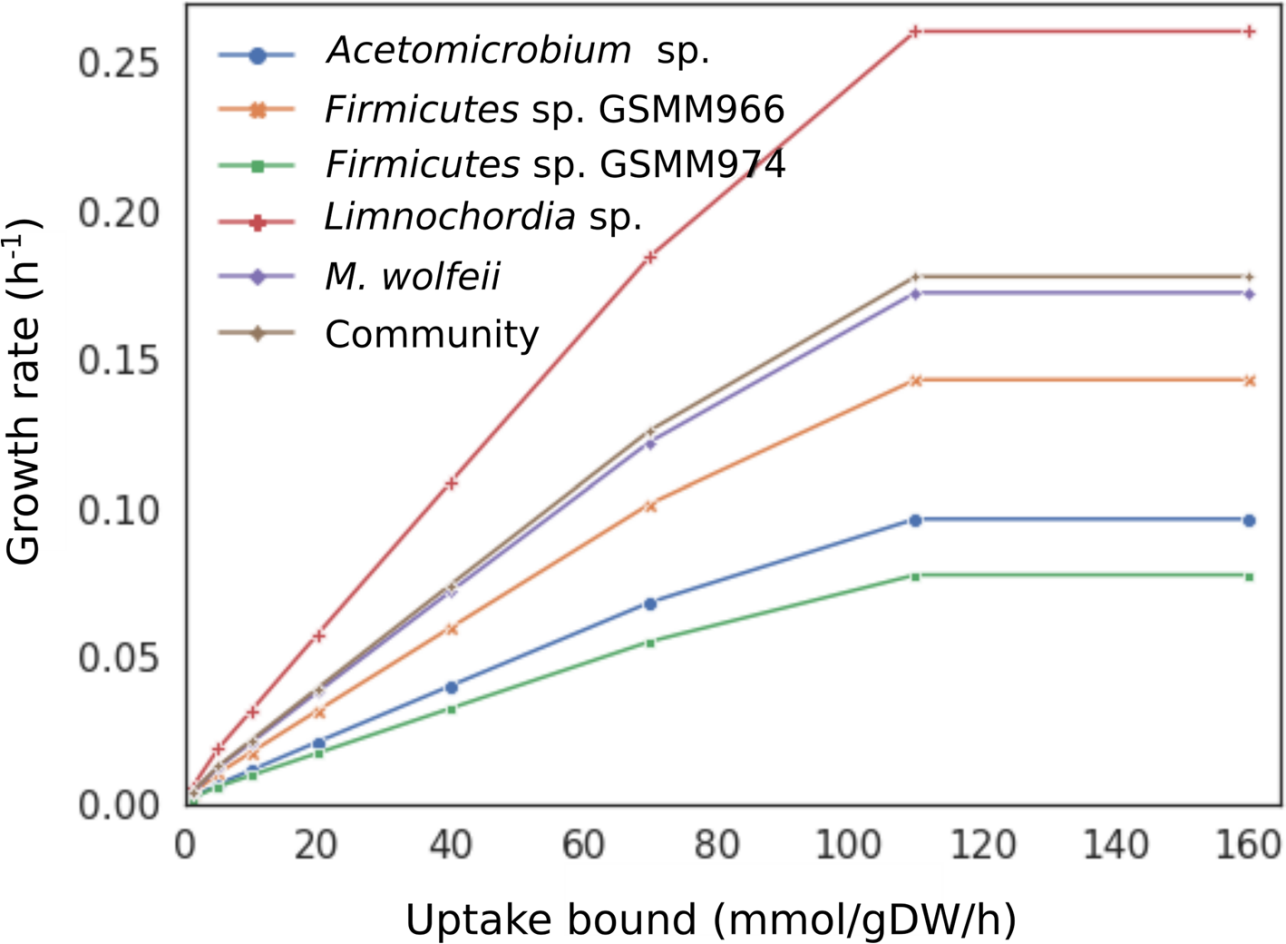
Species-specific and overall growth rate of the community predicted using different upper bounds set up for H_2_ and CO_2_ uptake rate. Growth rates were simulated using nine different upper bounds thresholds ranging from 0 to 160 mmol/gDW/h

To predict the microbial behavior under the applied experimental conditions, FBA was applied to a comprehensive metabolic network model of the community. This model composed by the GSMMs of the five dominant species was constrained with the available biochemical data and integrated with relative abundances (see “Materials and methods” section). Moreover, CO_2_ was the main carbon source included in the simulated growth medium. The strategy used to validate the community model was to assess the growth rate response of each member to the increasing uptake bounds of CO_2_ and H_2_ (Fig. 5).

By increasing the nutrient/compound availability it was possible to highlight the direct dependence of the species on the CO_2_/H_2_. The results showed that *Limnochordia* sp. GSMM975 and *M. wolfeii* GSMM957 had the highest proportional increase in growth rate with respect to the increasing uptake. These two species were expected to have the highest metabolic potential to exploit CO_2_, considering their dominance in the biofilm microbiome. Moreover, the overall community growth rate increased and reached a plateau at 0.18 h^−1^, whereas the individual growth rates appear proportional to taxon abundances, which agrees with trends highlighted by Diener et al. [38]. To further assess how the metabolic potential of species could affect their ability to consume CO_2_, the completeness of WLP, RGP and GSRP were evaluated. None of the MAGs have genetic evidence for the complete WLP but the gap-filling procedure added almost all its reactions in *Limnochordia* sp. GSMM975 and *Firmicutes* sp. GSMM966 (Supplementary material). When assessing completeness of alternative CO_2_ reduction pathways, *Acetomicrobium* sp. GSMM972 was the only MAG with a single missing reaction (i.e., aminomethyltransferase). Gap-filling and manual curations completed the RGP and GSRP in both *Acetomicrobium* sp. GSMM972 and *Limnochordia* sp. GSMM975, confirming their potential to utilize CO_2_ as a carbon source.

**Fig. 6:**
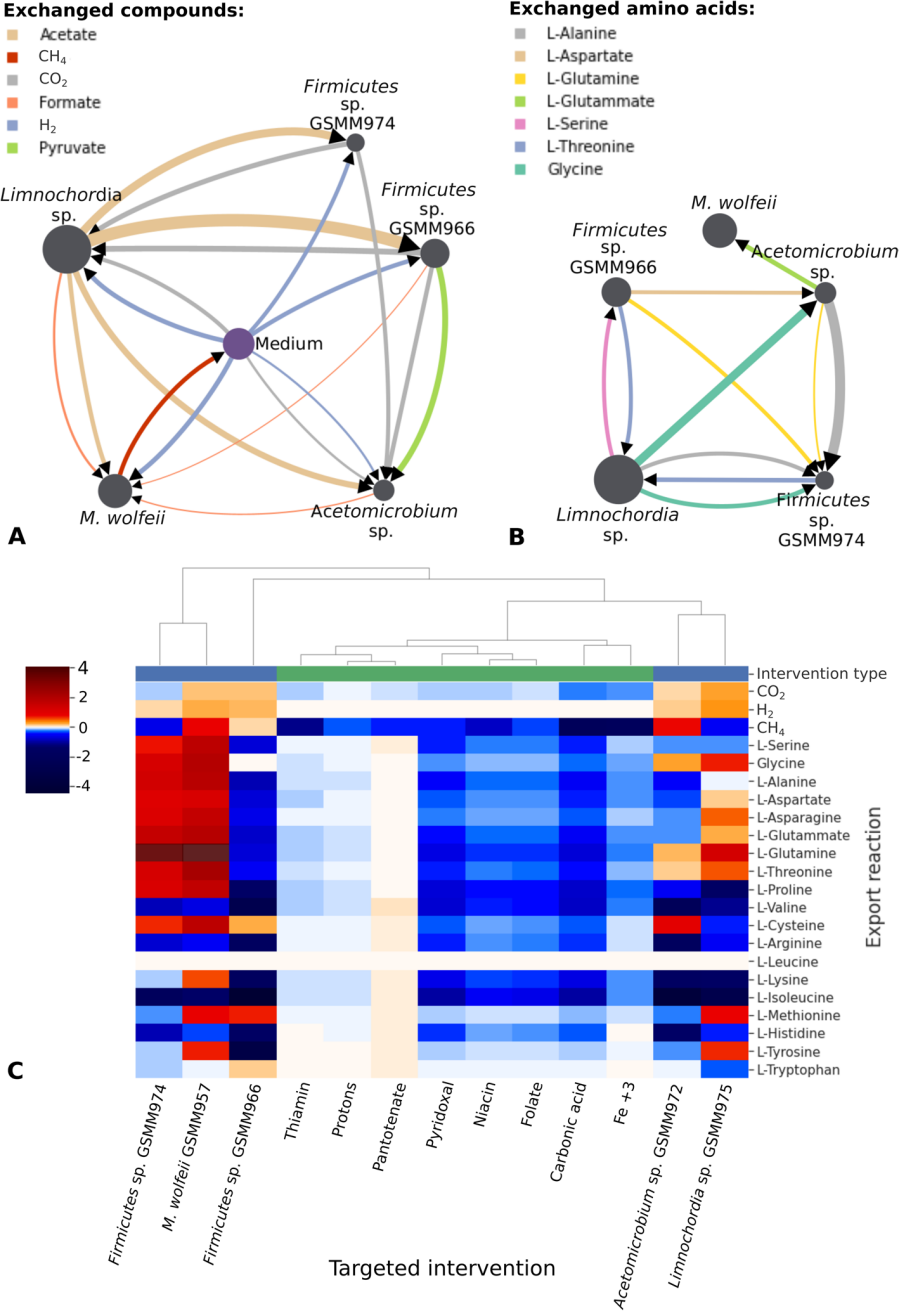
**A**, **B** Visualization of metabolic exchanges occurring between the most abundant species present in the biofilm and/or the medium. The purple circle represents the medium and grey circles the most abundant MAGs. The circle diameter is proportional to the microbe average relative abundance in the four biofilm samples. Results for key compounds related to biogas upgrading process (**A**) and all exchanged amino acids (**B**) are reported. Arrows thickness is proportional to the fluxes between species. **C** Effects of changes operated on feedstock and microbes’ abundance on amino acids, H_2_, CO_2_, and CH_4_ production. Targeted intervention on feedstock components or microbe species are in columns and the influenced export reactions in rows. Colors refer to elasticity, which is the change in metabolites production determined by an increase in the specific effector. Red/blue colors denote changes that would respectively increase/decrease the metabolite production. Only targeted intervention with nonzero elasticity for at least one of the selected compounds are reported. The types of intervention tested are divided in shifts of metabolites availability in green and species abundance in blue

Model simulation results depict the most relevant cross-feeding interactions, not easily measurable in vivo (Supplementary material); two subsets were represented as networks (Fig. 6). It is possible to notice that all the MAGs import H_2_ but, as expected, CH_4_ production is exclusively assigned to *M. wolfeii* GSMM957 being the only archaeon in the modeled community. Surprisingly, this species is not the largest consumer of CO_2_, which is mainly uptake by *Limnochordia* sp. GSMM975. As a consequence, only a fraction of the dissolved CO_2_ undergoes a direct conversion to CH_4_, whereas the majority of the gas is converted into some form of electron donors (e.g., formate) and acetate. In the community model, volatile fatty acids (VFA) accumulation in the medium was constrained to zero, mirroring the stable concentration measured experimentally (Supplementary material). Nevertheless, interspecies exchanges were predicted for acetate, which is produced by *Limnochordia* sp. GSMM975 to support the growth of the other MAGs (Fig. 6A). Propionate and butyrate were also predicted to be among the exchanged metabolites. The first is produced by both *Firmicutes* species and absorbed by *Acetomicrobium* sp. GSMM972, whereas the second is exported by *Firmicutes* sp. GSMM966 and supported the growth of *Limnochordia* sp. GSMM975.

The amino acids involved in cross-feeding among members of the microbial community are l-Glutamate, l-Aspartate, l-Glutamine, l-Serine, l-Threonine, l-Alanine, and glycine (Fig. 6B). By observing the predicted fluxes, the species shown to have paired reciprocal exchanges are *Limnochordia* sp. GSMM975 with *Firmicutes* sp. GSMM974 and GSMM966. In both cases, *Limnochordia* sp. GSMM975 imports l-Threonine, but on the first exchange it exports L-Alanine and glycine, and on the second l-Serine. Both l-Serine, glycine, and l-Alanine are precursors of pyruvate, an important compound of the TCA cycle and, therefore, crucial for energy gain purposes. A recent publication has evidenced the role of gluconeogenic aminoacids exchange in filling up the TCA cycle in the AD community [21]. *Limnochordia* sp. GSMM975 exports also a high amount of glycine headed for *Acetomicrobiom* sp. GSMM972, which is probably able to convert it into more energetically expensive amino acids. Moreover, it is interesting to notice that the only species predicted to exchange consistent amounts of amino acids with *M. wolfeii* GSMM957 is *Acetomicrobium* sp. GSMM972, which can synthesize l-Alanine, l-Glutamine, and l-Glutamate. Analyzing only amino acid exchanges, *Acetomicrobium* sp. GSMM972 appeared to play an important role for *Firmicutes* sp. GSMM974, which imports l-Alanine and l-Glutamine, and hypothetically establishes a facultative syntropy with *M. wolfeii* GSMM957, since it is the only species providing l-Glutamate.

Finally, the impact of targeted interventions on the net microbial consumption or production of amino acids, H_2_, CO_2_, and CH_4_ by the biofilm community was quantified. The types of interventions applied were increasing the availability of single metabolites in the feedstock or increasing the abundance of single microbial species. It is possible to observe that the most common effect of these perturbations is to diminish the overall amino acid and CH_4_ production (Fig. 6C). Specifically, all the modifications to the availability of metabolites negatively affect almost all the production of amino acids and CH_4_. On the contrary, the interventions in species abundance have more heterogeneous consequences. Regarding *Firmicutes* sp. GSMM966 and *Limnochordia* sp. GSMM975, modification in abundances had a general negative impact on amino acid and CH_4_ production. The only notable exception is L-Tyrosine, whose export doubled when the abundance of *Limnochordia* sp. GSMM975 was increased. However, the increased abundance of the other three species has a general positive effect on CH_4_ and amino acid production. The fundamental role of *M. wolfeii* GSMM957 *was* confirmed in the community since its increase significantly improves both the production of CH_4_ and most amino acids. Interestingly, the intervention in *Acetomicrobium* sp. GSMM972 abundance has a positive effect on CH_4_ production as well, which may confirm the positive interplay with *M. wolfeii* GSMM957, suggested by the previously mentioned correlation coefficient (Fig. 2B).

## Discussion

Integration of metagenomics binning and metabolic modelling represent a cutting-edge method to study microbial communities involved in CH_4_ production. In the current study, a simple biofilm community was investigated to disentangle the microbial dynamic characteristics of engineering AD systems. Analysis showed a low complexity metagenome (59 MAGs) with five MAGs accounting for over 70% of the community abundance in each reactor. The comparisons of the recovered MAGs with the MiGa Biogas microbiome database demonstrates that several species reciprocally co-occur at a statistically significant level; this is suggesting the existence of a core set of species that potentially establish mutually beneficial interactions. Regarding the species role that can be inferred from their relative abundance, *Limnochordia* sp. GSMM975 was found as the dominant MAG in several full and laboratory scale biogas reactors operating at thermophilic condition [36]. In the work of Treu at al. (2018), the relative abundance of this species substantially increased after hydrogenation, suggesting its role as homoacetogen [49]. In a recent study, the AD microbial community was gradually simplified by reducing the carbon source diversity, to reveal numerosity and cross-feeding interactions of the keystone species [7]. *Limnochordia* sp. GSMM975 (previously named as *Firmicutes* sp. DTU0495 in the cited study) was found to adapt to simple carbon sources in two out of four tested conditions (i.e., a mixture of VFA and the sole acetate). The ability of this bacterium to consume acetate was furthermore corroborated by the application of protein-stable isotope probing under simulation of acetate accumulation conditions [50]. Similarly, *Limnochordia* sp. played a significant role in adaptation to high ammonia levels with chemically defined substrates as energy source (i.e., methanol, acetate, formate, CO_2_, and H_2_) [18]. In this latter work, *Limnochordia* sp. GSMM975 was identified as one of the dominant members in most of the different nutritional conditions. Moreover, a co-occurrent hydrogenotrophic archaeon, *M. wolfeii* DTU779, was proposed as the syntrophic partner of *Limnochordia* sp. GSMM975 in two of the tested conditions. Nevertheless, despite the very high abundance of *Limnochordia* sp. GSMM975 in reactors R2 and R3 (~ 33%), this microbe is not the only putative syntrophic species of the methanogens; in fact, other members of the community (e.g., *Acetomicrobium* sp. GSMM972 and *Tepidanaerobacter* sp. GSMM928) are probably able to provide benefits and support the growth of *M. wolfeii* [51]. For example, species of *Acetomicrobium* genus are known to be involved in hydrolysis and acidogenesis, producing acetate, H_2_ and CO_2_ from sugar fermentation and VFA degradation [52]. These byproducts of the fermentation process could support the growth of methanogens, justifying the similarities identified in the coverage profiles (*r* = 0.71). Moreover, since the high H_2_ concentration influences the thermodynamic feasibility of acidogenic reactions, the syntrophic interaction between *Methanothermobacter* spp. and homoacetogens was highlighted in other similar environments [5].

In addition, the high relative abundances of the five species under evaluation suggest that the close proximity due to embedding within the biofilm had a positive effect on species fitness. Previous studies also demonstrated that biofilms in biogas reactors lead to more efficient and stable consumption of organic substrates and increased the amount of CH_4_ produced [44]. A possible explanation to this phenomenon is that EPS secretion by microorganisms generates an embedding material crucial for the development of a protected growth environment [53]. Moreover, the advantages derived from microbes being able to coexist in complex aggregates formation have been associated with the improvement of metabolic exchanges which foster the establishment of syntrophic interactions [46]. A second reason that could have helped the dominant species to prevail was the spatial localization of the biofilm around the diffusers which guaranteed a higher H_2_ and CO_2_ availability. Even though it cannot be disputed that all the five dominant microbes might have a direct or indirect role in biofilm formation, the results from the current work provide solid proofs for *Firmicutes* spp. GSMM966, GSMM974, and *Limnochordia* sp. GSMM975 to be the main species potentially involved in the EPS biosynthesis. *Firmicutes* spp. GSMM966 and GSMM974 demonstrate also to have protein secretion system and pilus assembly genes, which may be relevant in the biofilm generation process. Pili in fact play an important role in the adherence of microorganisms, aiding in the colonization of ecological niches through the formation of biofilms. In particular, type II/IV pili, are involved in a form of motility known as twitching, which is essential for biofilm formation and contributes to mediate adherence to surfaces [54, 55]. In addition, all the dominant species are potentially able to synthesize spermidine that, in *Bacillus subtilis*, has been proven not essential for normal planktonic growth, but necessary for robust biofilm formation [56, 57]. In other cases (i.e., in *Vibrio cholerae*) spermidine can also enhance biofilm formation via multiple signaling pathways [58]. Within the reactor system, the establishment of the biofilm introduced an additional level of complexity determined by the interaction between subsystems (i.e., planktonic, and fixed microbial communities). This relationship has not been analyzed in the current work; however, it is remarkable to notice that some of the prevalent species previously found in the liquid phase are the most abundant also in the biofilm [27]. The ubiquity of species in the systems, combined with the fact that microbial consortia immobilized in biofilms are not affected by dilution processes, can suggest that these mucous structures actually play an active role in shaping the planktonic community. The knowledgebase of mechanisms and roles of species involved in EPS formation is therefore fundamental in order to be able to influence methanogenic consortia and increase CH_4_ yield in AD process [59].

Further predictions on metabolic potential of the dominant microbes were performed through genome-guided metabolic reconstruction strategies. Results demonstrate that the reconstructed metabolic network of *M. wolfeii* GSMM957 includes, as expected, hydrogenotrophic-specific modules and is able to synthesize essential coenzymes (i.e., M and F420) and intermediates (i.e., PRPP) involved in the methanogenesis. Among the other predicted features, *M. wolfeii* GSMM957 exhibits almost complete prototrophy for amino acids but it has a simplified central carbon metabolism. Vice versa, *Limnochordia* sp. GSMM975 has numerous auxotrophies for amino acids that could highlight the existence of syntrophic associations with other microbes in the biofilm. Indeed, the bacterium encodes multiple amino acid transporters and some enzymes involved in specific degradation pathways (i.e., histidine). However, the genome of *Limnochordia* sp. GSMM975 presents many other functional pathways which indicates its potential activity in different steps of the AD food chain. Two of them, worth to be mentioned, include the conversion of carbohydrates directly into substrates used by the archaeon and the glycine reductive pathway necessary for autotrophic growth on CO_2_. Therefore, due to the versatile metabolism of this species, it is possible that this microbe can perform hydrolysis of complex substrates, thus occupying multiple layers in the so-called anaerobic digestion funnel [59]. These multifunctional roles would explain why the species is often present in the microbial community of different biogas plants, and why it is more ubiquitous compared to other known SAOB [60, 61]. Moreover, the complementarity of the metabolisms of *Limnochordia* sp. GSMM975 and *M. wolfeii* GSMM957 and the frequent co-occurrence of these two microbial species in the scrutinized AD samples helped to support the hypothesis of facultative syntrophic relationship between the two.

Furthermore, insight of the relationship between them comes from in situ hybridization analysis. Results from FISH clearly highlight the co-localization of the taxa within the same flocs. This observation of *Limnochordia* sp. GSMM975 and *M. wolfeii* GSMM957 acclimatized in simple BA medium support their connection and corroborates the hypothesis about their reciprocal benefit during CO_2_ methanation. Indeed, it has been previously demonstrated that a reduced interspecies distance in complex ecosystems is advantageous for methanogens in syntrophic consortia, due to higher electron transfer efficiency [62].

A tentative connection between microbial community composition and ecosystem function was established in order to define a customizable metabolic model of the AD microbiome. This approach allowed a detailed view of the behavior of the community under the applied experimental conditions, and the evaluation of the metabolites exchanged between MAGs. As expected, the simulation showed that H_2_ can be imported by *M. wolfeii* GSMM957, which is consistent with hydrogenotrophic methanogens being known to use mainly H_2_ and formate as interspecies electron transfer compounds in syntrophic relations [63]. Nonetheless, it is possible to notice that all the other MAGs also import H_2_, suggesting that they too have metabolic pathways involved in the utilization of H_2_ as electron donor. This behavior agrees with the fact that the biofilm was generated on the surface of the CO_2_/H_2_ diffusers and membranes in the reactor, the sites where the concentration of dissolved gases is expected to be the highest. Results showed also that *Limnochordia* sp. GSMM975 was consuming most of the available CO_2_, reducing much of it into acetate. However, despite the apparent competition for the main carbon source between the two microbes, the establishment of a syntrophic relationship may depend on intermediate electron donors. In fact, *Limnochordia* sp. GSMM975 was proposed to convert CO_2_ into formate, subsequently consumed by *M. wolfeii* GSMM957. In the simulation, *M. wolfeii* GSMM957 is the only importer of formate, and this result was also confirmed by the coverage profile analysis, where it was found to have ~ 7% relative abundance in an experiment using BA medium for growth and formate as carbon source [18]. Additionally, a previous study showed that formate was effectively used as the sole carbon source supporting the growth of *M. wolfeii* [64].

The interactions between microbes have been investigated also in terms of exchange of more complex metabolites, such as amino acids and VFA. From the analysis emerged that *Acetomicrobium* sp. GSMM972 is able to use acetate and propionate to grow. Although degradative capacities have not been demonstrated yet, a previous study showed that the genus *Acetomicrobium* was enriched in cultures fed only with acetate or propionate [65]. Therefore, the new putative degrading properties associated with *Acetomicrobium* sp. GSMM972 may justify its abundance under the described conditions. This species appeared also to have a role in amino acids synthesis for other members of the community. For example, it hypothetically established a facultative syntropy with *M. wolfeii* GSMM957 based on L-Glutamate exchange. Also, the predicted capacity of *Limnochordia* sp. to exchange amino acids adds to the evidence that this species has a very versatile metabolism and can cooperate through different mechanisms, thus, explaining its central role in AD communities. However, it should be noted that, since the metabolic models of MAGs were individually gap-filled using minimal medium lacking amino acids, the identity and size of truly exchanged amino acids among species is probably overestimated. This happens, because during the first step of gap-filling, the algorithm adds all those missing reactions to the GSMM, which are essential to guarantee biomass production (i.e., in silico growth) under a given nutritional landscape. Nevertheless, the central role of amino acids in cooperative communities has been already suggested in aerobic and anoxic environments; they have been proposed as a mechanism to fill the tricarboxylic acid cycle [21, 66]. Indeed, many amino acids are the precursors of compounds belonging to the TCA cycle, as for example the aforementioned L-Serine and l-Alanine that are precursors of pyruvate [67]. Furthermore, l-Aspartate and l-Asparagine are precursors of oxaloacetate [68]. Finally, due to the importance of hydrolytic bacteria in the AD funnel, amino acids have shown to play a crucial role in the establishment of the interaction networks in AD communities.

## Conclusions

In the present work, a comprehensive investigation combining genome-centric metagenomics and flux balance analysis was used to inspect a microbiota responsible for CO_2_ methanation during the biogas upgrading process. The obtained findings unraveled pathways involved in exopolysaccharide biosynthesis and the syntrophic relationships shaping the microbiome, including cross-feedings between species. *Limnochordia* sp. GSMM975 and *M. wolfeii* GSMM957 were identified as very abundant and correlated in a wide set of experimental conditions; *Firmicutes* sp. GSMM966 was specifically linked to the exopolysaccharides production. Genome-scale models were used to simulate the metabolism and the cross-feedings occurring in the microbiome. Moreover, constraints-based models’ implementation is an important addition to the methodologies currently in use for microbial ecology. In particular, the five dominant species can be considered as facultative syntrophs; thus, the network of interactions showed a degree of elasticity where some of them are ubiquitous (*Limnochordia* sp. GSMM975 and *Acetomicrobium* sp. GSMM972) while others have a scattered distribution (*M. wolfeii* GSMM957). *Limnochordia* sp. GSMM975 and *Acetomicrobium* sp. GSMM972 have a very complex metabolism and form a relationship mainly based on metabolite exchanges. Ultimately, it is concluded that an efficient collaboration based on amino acids exchanges between the diverse species is essential in maintaining a stable process. Considering the relevance of biofilm formation in several reactor configurations (i.e., trickle bed, membrane-based), the presence of these five microbes in the inoculum would be considered as a desired feature. Indeed, with the recent developments in the microbiological field, the opportunity to apply FBA to examine species interactions aids a rational modification of communities playing a role in biotechnological, precision agriculture or bioremediation strategies.

## Supplementary Information

**Additional file 1. Supplementary material. **The genomes of the two most abundant species were independently assembled, their scaffolds were ordered by manual curation, and their closed chromosomes were drawn using Artemis [1]. The same software (parameters: windows size 10,000 bp and step size 200 bp) was also used to determine the GC content and the GC skew. The COG annotation was converted to “EMBL” format using in-house Perl scripts and added to the graphical representation in “gff” format reporting the gene positions predicted by Prodigal. COG codes assigned to the proteins were used to color the genes (https://www.ncbi.nlm.nih.gov/research/cog). The reconstruction of the five most abundant species metabolisms were also performed to investigate their role within the community. The reconstruction was performed from KEGG codes obtained using eggNOG mapper. The resulting networks were visually inspected in KEGG database using KEGG Mapper – Color Pathway, and results are depicted in Figures S5-S9. **Table S1**. Reactors pH, VFA, dissolved H_2_ concentration (H_2l_) and H_2_ transfer coefficient (k_L_a) under steady state conditions at period IV. Descriptive biochemical data of the analyzed phase were collected in a previous work and are reported here [2]. **Table S2**. Reactors' performances under steady state conditions at period 6. Q_OUT_ represents the total gas output rate [L/L_R_.d] and Q_OUT,T_ the total theoretical gas output rate [L/L_R_.d]. As described by Bassani et al. P_CH4_ indicates the CH_4_ production rate, Y_CH4_ the CH_4_ yield, n_H2_ and n_CO2_ the hydrogen and CO_2_ conversion efficiency, respectively [2]. **Figure S1**. Representation of the reconstructed genome of *Methanothermobacter wolfeii* DTU-UNIPD957*. ***Figure S2**. Representation of the reconstructed genome of *Lymnochordia* s*p. GSMM975. ***Figure S2**. Representation of the reconstructed genome of *Lymnochordia* s*p. GSMM975. ***Figure S4**. Overall import and export fluxes (> 1 mmol/gDW/hr) of the community from the environment. **Figure S5**. Metabolic reconstruction of Acetomicrobium sp. GSSM972. **Figure S6**. Metabolic reconstruction of Methanothermobacter wolfeii GSSM957. **Figure S7**. Metabolic reconstruction of Firmicutes sp. GSSM974. **Figure S8**. Metabolic reconstruction of Firmicutes sp. GSSM966. **Figure S9**. Metabolic reconstruction of Limnochordia sp. GSSM975. **Figure S10**. Reactions and corresponding genetic evidence of CO_2_ reduction pathways included in the GSMM. **Figure S11**. FISH image of the microbial community.

**Additional file 2: Dataset S1**. Genes responsible for EPS biosynthesis or involved in biofilm formation according to Model Seed categories used for targeted investigation through Hidden Markov Models. List of KEGG code, reaction and name of genes searched, combined with the number of copies found in each genome. “*” refers to results retrieved by EggNOG annotation alone, “^” refer to results obtained through HMM search and no annotation refers to results retrieved by RAST.

**Additional file 3: Dataset S2**. Metagenomes clusters used to summarize the abundance profile of MAGs and experiment related unique identifiers of raw reads. The corresponding reference is associated with each experiment used for coverage profile calculation.

**Additional file 4: Dataset S3**. KEGG modules completeness level of MAGs. Integrated results of EggNOG and Diamond annotations.

**Additional file 5: Dataset S4**. Genome scale metabolic models reconstructed for the five most abundant species and their quality assessment. Models are available in SBML format, and the reports generated with MEMOTE in HTML format at 10.6084/m9.figshare.16692028.

**Additional file 6: Dataset S5**. Basic Anaerobic Medium composition used for the community model simulation. “Compounds” are the unique ModelSEED identifier, “Name” the corresponding human readable metabolites and “MaxFlux” the uptake upper bound allowed form the feedstock.

**Additional file 7: Dataset S6**. MAG statistics and phylogenetic classification. General information regarding the recovered 59 MAGs.

**Additional file 8: Dataset S7**. Operons predicted by Operon Mapper in MAGs. Results are available at 10.6084/m9.figshare.16691878.

**Additional file 9: Dataset S8**. Illumina filtered reads and raw nanopore reads quality assessment. Results obtained with FastQC (v.0.11.9) and NanoPlot (v.1.32.1) are available at 10.6084/m9.figshare.19355303.

## Data Availability

All the sequences of this study have been submitted to the National Center for Biotechnology Information (NCBI) under the project BioProject PRJNA762202. Illumina filtered reads and raw nanopore reads quality assessment can be found in Supplementary Dataset S8.

## Abbreviations

AD: Anaerobic digestion
CO_2_: Carbon dioxide
CH_4_: Methane
H_2_: Hydrogen
SAOB: Syntrophic acetate oxidizing bacteria
WLP: Wood-Ljungdahl pathway
EPS: Extracellular polymeric substances
MAGs: Metagenome-assembled genomes
GSMM: Genome scale metabolic models
FBA: Flux balance analysis
FISH: Fluorescence in situ hybridization
BA: Basal anaerobic medium
VFA: Volatile fatty acids
CDSs: Coding DNA sequences
GCS: Glycine cleavage system
RGP: Reductive glycine pathway
