# Supplementary material for: Integrating metagenomic binning with flux balance analysis to unravel syntrophies in anaerobic CO2 methanation
Source: Microbiome. 2022 Aug 3;10:117. doi: 10.1186/s40168-022-01311-1 (PMC9347119; doi:10.1186/s40168-022-01311-1)
Supplement: Supplementary file 2 — Additional file 1. Supplementary material. The genomes of the two most abundant species were independently assembled, their scaffolds were ordered by manual curation, and their closed chromosomes were drawn using Artemis [1]. The same software (parameters: windows size 10,000 bp and step size 200 bp) was also used to determine the GC content and the GC skew. The COG annotation was converted to “EMBL” format using in-house Perl scripts and added to the graphical representation in “gff” format reporting the gene positions predicted by Prodigal. COG codes assigned to the proteins were used to color the genes (https://www.ncbi.nlm.nih.gov/research/cog). The reconstruction of the five most abundant species metabolisms were also performed to investigate their role within the community. The reconstruction was performed from KEGG codes obtained using eggNOG mapper. The resulting networks were visually inspected in KEGG database using KEGG Mapper – Color Pathway, and results are depicted in Figures S5-S9. Table S1. Reactors pH, VFA, dissolved H2 concentration (H2l) and H2 transfer coefficient (kLa) under steady state conditions at period IV. Descriptive biochemical data of the analyzed phase were collected in a previous work and are reported here [2]. Table S2. Reactors' performances under steady state conditions at period 6. QOUT represents the total gas output rate [L/LR.d] and QOUT,T the total theoretical gas output rate [L/LR.d]. As described by Bassani et al. PCH4 indicates the CH4 production rate, YCH4 the CH4 yield, nH2 and nCO2 the hydrogen and CO2 conversion efficiency, respectively [2]. Figure S1. Representation of the reconstructed genome of Methanothermobacter wolfeii DTU-UNIPD957. Figure S2. Representation of the reconstructed genome of Lymnochordia sp. GSMM975. Figure S2. Representation of the reconstructed genome of Lymnochordia sp. GSMM975. Figure S4. Overall import and export fluxes (> 1 mmol/gDW/hr) of the community from the environment. Figure S5. Metaboli [file 40168_2022_1311_MOESM1_ESM.docx]

**Integrating metagenomic binning with flux balance analysis to unravel syntrophies in anaerobic CO_2_ methanation**

De Bernardini N.^1^, Basile A.^1^, Zampieri G.^1^, Kovalovszki A.^2^, De Diego Diaz B.^3^, Offer E.^1^, Wongfaed N.^4^, Angelidaki I.^5^, Kougias P.G.*^,6^, Campanaro S.*§^1,7^, Treu L.§^1^

^1^Department of Biology, University of Padova, Via U. Bassi 58/b, 35121 Padua, Italy

^2^Department of Environmental Engineering, Technical University of Denmark, 2800 Kgs. Lyngby, Denmark.

^3^Department of Chemistry, University of Navarra, Spain

^4^Department of Biotechnology, Faculty of Technology, Khon Kaen University, Khon Kaen 40002, Thailand

^5^Department of Chemical and Biochemical Engineering, Technical University of Denmark, Kgs., Lyngby DK-2800, Denmark

^6^Hellenic Agricultural Organization DEMETER, Soil and Water Resources Institute, Thermi-Thessaloniki, Greece.

^7^CRIBI Biotechnology Center, University of Padova, 35131 Padua, Italy.

^*^Corresponding authors: Campanaro S., Kougias P.G.

E-mail address: stefano.campanaro@unipd.it;

[p.kougias@swri.gr](mailto:p.kougias@swri.gr);

### **Supporting information includes:**

Materials and methods

Table S1 to S2

Figures S1 to S11

Datasets S1 to S7

References

####

#### **Materials and methods**

The genomes of the two most abundant species were independently assembled, their scaffolds were ordered by manual curation, and their closed chromosomes were drawn using Artemis [1]. The same software (parameters: windows size 10,000 bp and step size 200 bp) was also used to determine the GC content and the GC skew. The COG annotation was converted to “EMBL” format using in-house Perl scripts and added to the graphical representation in “gff” format reporting the gene positions predicted by Prodigal. COG codes assigned to the proteins were used to color the genes (<https://www.ncbi.nlm.nih.gov/research/cog>).

The reconstruction of the five most abundant species metabolisms were also performed to investigate their role within the community. The reconstruction was performed from KEGG codes obtained using eggNOG mapper. The resulting networks were visually inspected in KEGG database using KEGG Mapper – Color Pathway, and results are depicted in Figures S5-S9.

#### **Table S1 to S3**

**Table S1. Reactors pH, VFA, dissolved H_2_ concentration (H_2l_) and H_2_ transfer coefficient (k_L_a) under steady state conditions at period IV.** Descriptive biochemical data of the analyzed phase were collected in a previous work and are reported here [2].

| Reactor | pH | TVFA  [g/L] | Acetate  [g/L] | Propionate  [g/L] | H_2l_  [M] | k_L_a  [day^−1^] |
| --- | --- | --- | --- | --- | --- | --- |
| R1 | 8.11 ± 0.15 | 0.17 ± 0.04 | 0.08 ± 0.02 | 0.06 ± 0.02 | (0.52 ± 0.10)*10^−6^ | 9.32*10^3^ |
| R2 | 8.03 ± 0.12 | 0.11 ± 0.07 | 0.07 ± 0.05 | 0.02 ± 0.03 | (0.23 ± 0.06)*10^−6^ | 15.21*10^3^ |
| R3 | 8.13 ± 0.17 | 0.31 ± 0.04 | 0.22 ± 0.05 | 0.05 ± 0.01 | (0.29 ± 0.03)*10^−6^ | 7.50*10^3^ |
| R4 | 8.09 ± 0.14 | 0.18 ± 0.07 | 0.13 ± 0.05 | 0.03 ± 0.01 | (0.22 ± 0.01)*10^−6^ | 7.47*10^3^ |

**Table S2. Reactors' performances under steady state conditions at period 6.** Q_OUT_ represents the total gas output rate [L/L_R_.d] and Q_OUT,T_ the total theoretical gas output rate [L/L_R_.d]. As described by Bassani et al. P_CH4_ indicates the CH_4_ production rate, Y_CH4_  the CH_4_ yield, n_H2_ and n_CO2_ the hydrogen and CO_2_ conversion efficiency, respectively [2].

| GRT  [h] | Q_RC_  [L/L_R_.h] | Reactor | Q_IN_  [L/L_R_.d] | Q_OUT,T_ [L/L_R_ .d] | Q_OUT_ [L/L_R_.d] | Output gas composition | | | P_CH4_ [L_CH4_ /L_R_d] | Y _CH4_ [L_CH4_ /L_H2_ ] | n H _2_ [%] | n CO _2_ [%] |
| --- | --- | --- | --- | --- | --- | --- | --- | --- | --- | --- | --- | --- |
|  |  |  |  |  |  | CH_4_ [%] | CO_2_ [%] | H_2_ [%] |  |  |  |  |
| 4 | 20.14 | R1 | 5.93±0.03 | 2.37±0.01 | 2.37±0.12 | 92.7±1.9 | 2.4±0.7 | 4.9±1.5 | 0.79±0.11 | 0.22±0.03 | 99.6±0.7 | 97.7±1.7 |
|  |  | R2 | 5.86±0.02 | 2.34±0.01 | 2.32±0.16 | 96.0±0.5 | 1.4±0.3 | 2.6±0.5 | 0.82±0.15 | 0.23±0.04 | 100.0±0.1 | 100.0±1.0 |
|  |  | R3 | 6.04±0.06 | 2.42±0.02 | 2.30±0.17 | 92.5±0.6 | 2.0±0.5 | 5.6±0.6 | 0.70±0.15 | 0.19±0.04 | 99.7±0.3 | 99.3±1.3 |
|  |  | R4 | 5.59±0.01 | 2.22±0.01 | 2.16±0.03 | 94.1±1.2 | 1.7±0.3 | 4.2±0.9 | 0.71±0.02 | 0.21±0.01 | 100.0±0.1 | 99.9±0.6 |

#### **Figures S1 to S10**


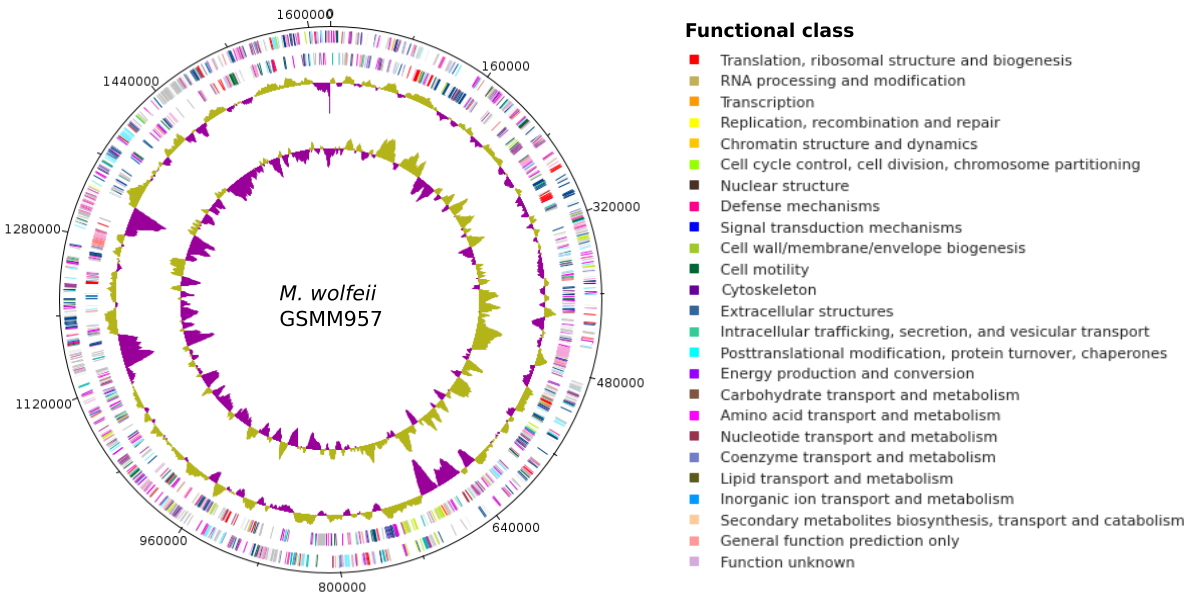


**Figure S1. Representation of the reconstructed genome of *Methanothermobacter wolfeii* DTU-UNIPD957*.*** Tentative reconstruction of the *Methanothermobacter wolfeii* DTU-UNIPD957 chromosome. The scaffolds obtained from the assembly and binning process were ordered taking into account the replication origin and the GC skew. From the inner to the outer concentric circles; circle 1, GC skew; circle 2, G+C content; circles 3 and 4, predicted protein-coding sequences (CDS) transcribed clockwise (outer part) and counter clockwise (inner part) and coloured according to the COG association.

**
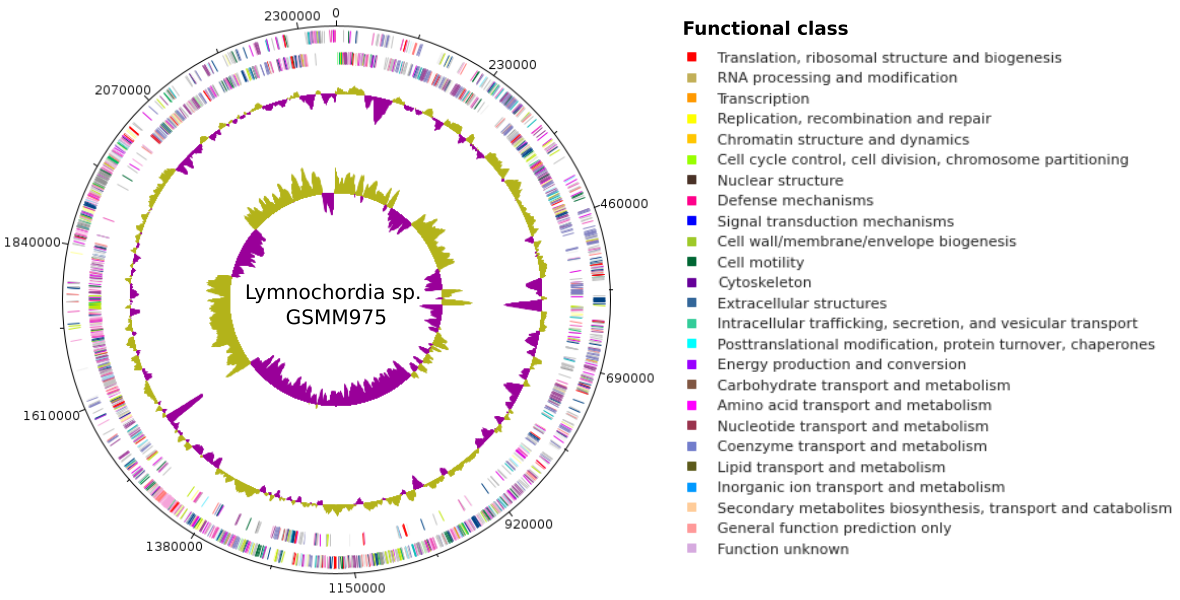
**

**Figure S2. Representation of the reconstructed genome of *Lymnochordia* s*p. GSMM975.*** Tentative reconstruction of the *Lymnochordia* sp. GSMM975 chromosome. The scaffolds obtained from the assembly and binning process were ordered taking into account the replication origin and the GC skew. From the inner to the outer concentric circles; circle 1, GC skew; circle 2, G+C content; circles 3 and 4, predicted protein-coding sequences (CDS) transcribed clockwise (outer part) and counter clockwise (inner part) and coloured according to the COG association (legend on the right part of the figure).


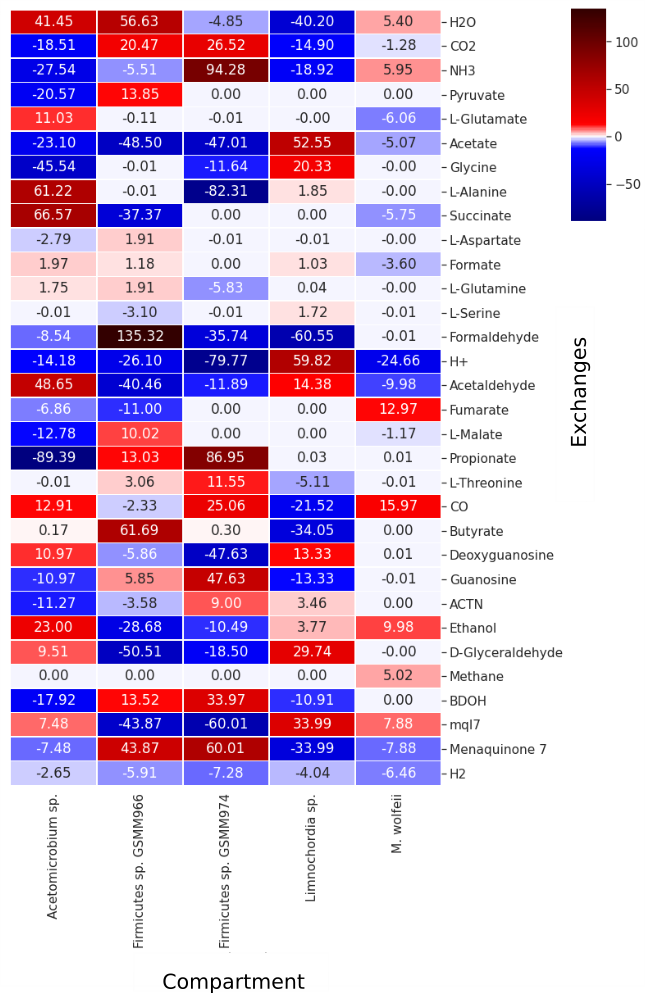


**Figure S3. Heatmap representing fluxes of selected compounds.** Production (red) and consumption (blue) capacities are reported for selected compounds and results are separated by MAG. Fluxes are shown in millimoles per hour per gram (dry weight) of the microbial community.


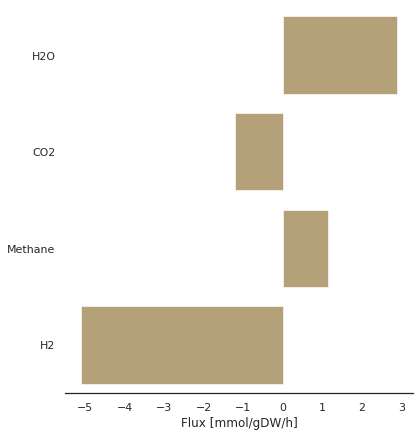


**Figure S4. Overall import and export fluxes ( > 1 mmol/gDW/hr) of the community from the environment.** The analysis indicates that the compounds consumed and accumulated in the growth medium are mainly H_2_O, CO_2_, H_2_ and methane. The upper and lower threshold of the flux values of CO_2_ and H_2_ and only the lower threshold of CH_4_ flux were constrained according to the bounds computed starting from the available biochemical data.

**
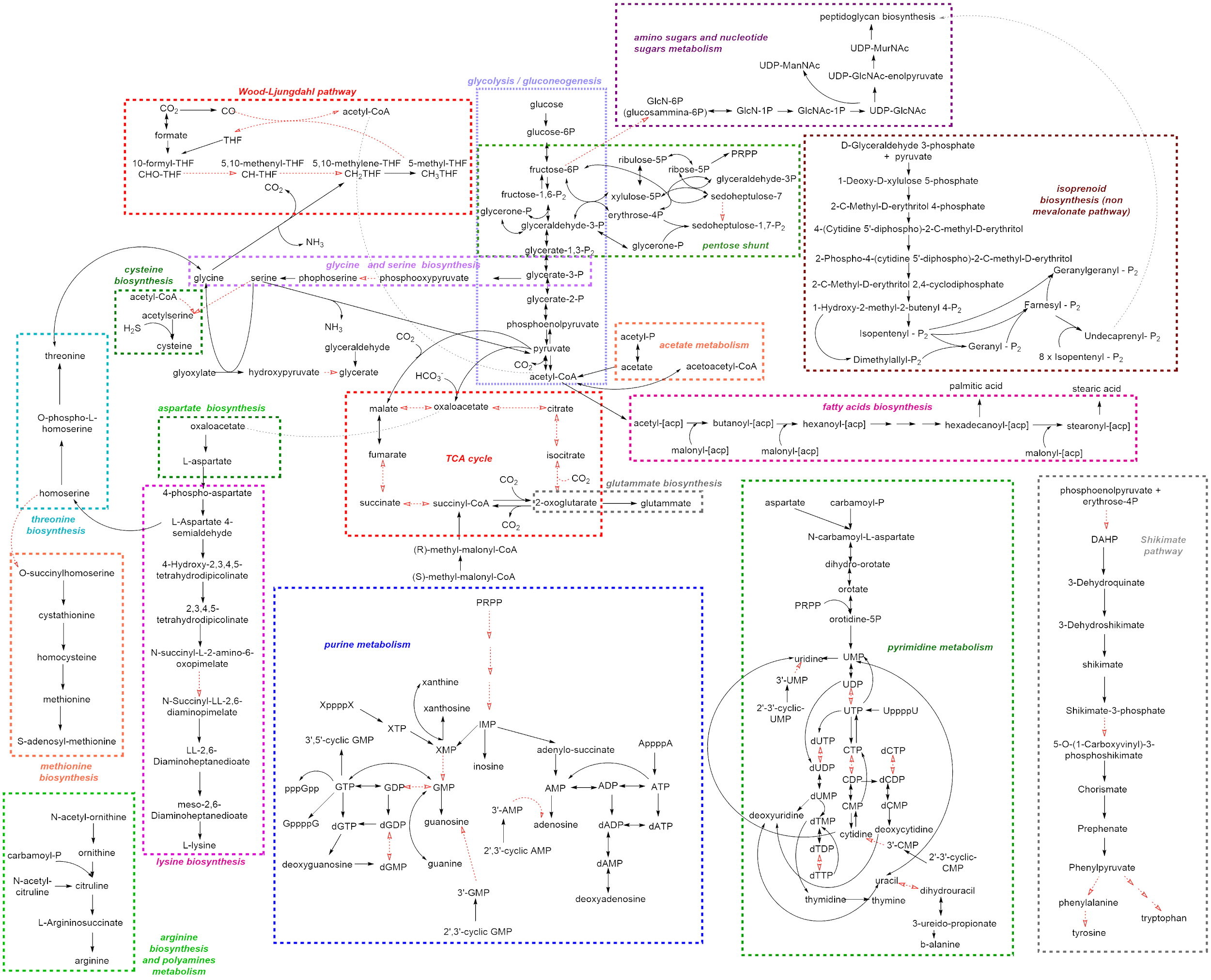
**

**Figure S5. Metabolic reconstruction of Acetomicrobium sp. GSSM972.**

**
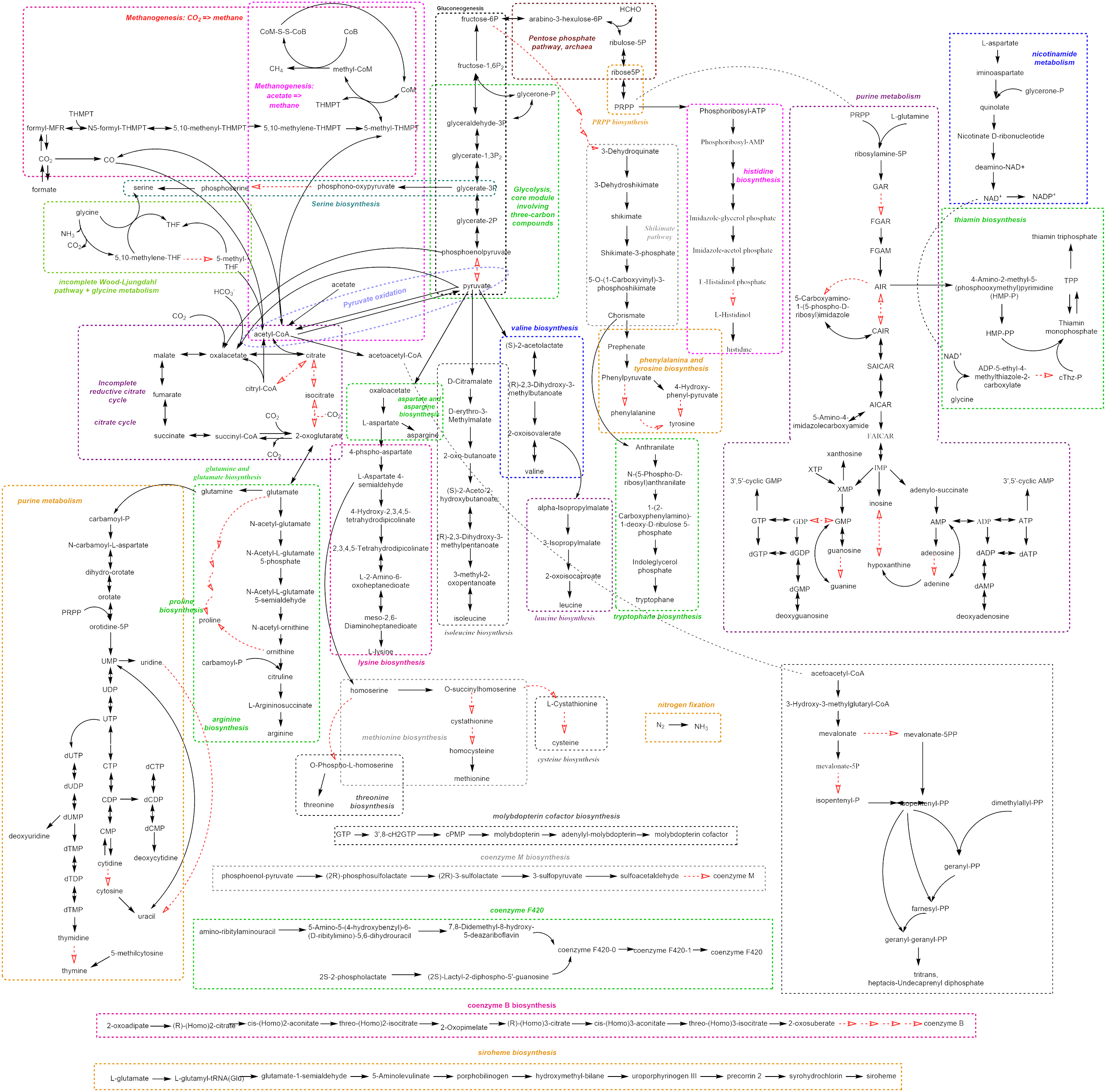
**

**Figure S6. Metabolic reconstruction of Methanothermobacter wolfeii GSSM957.**

**
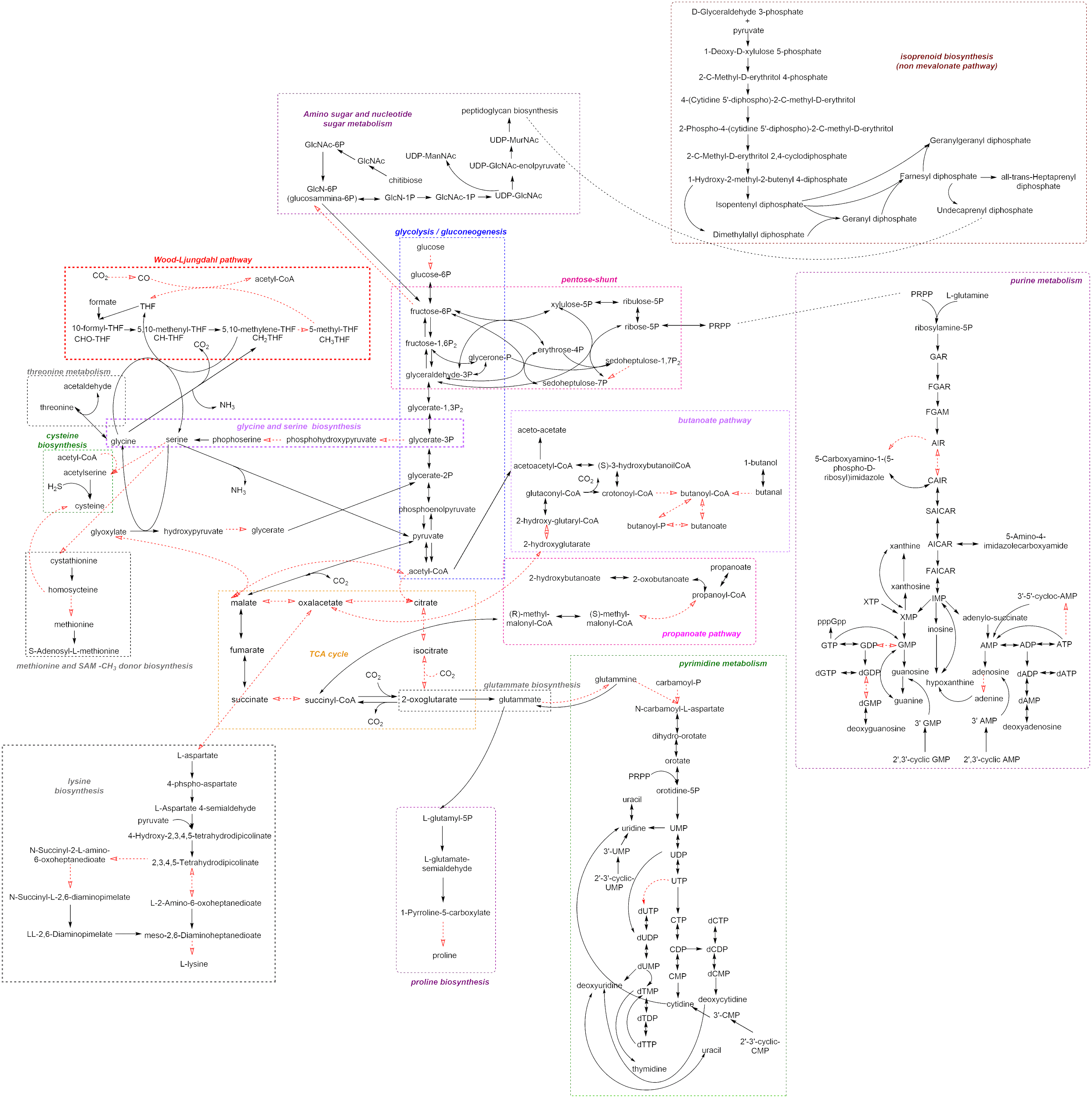
**

**Figure S7. Metabolic reconstruction of Firmicutes sp. GSSM974.**

**
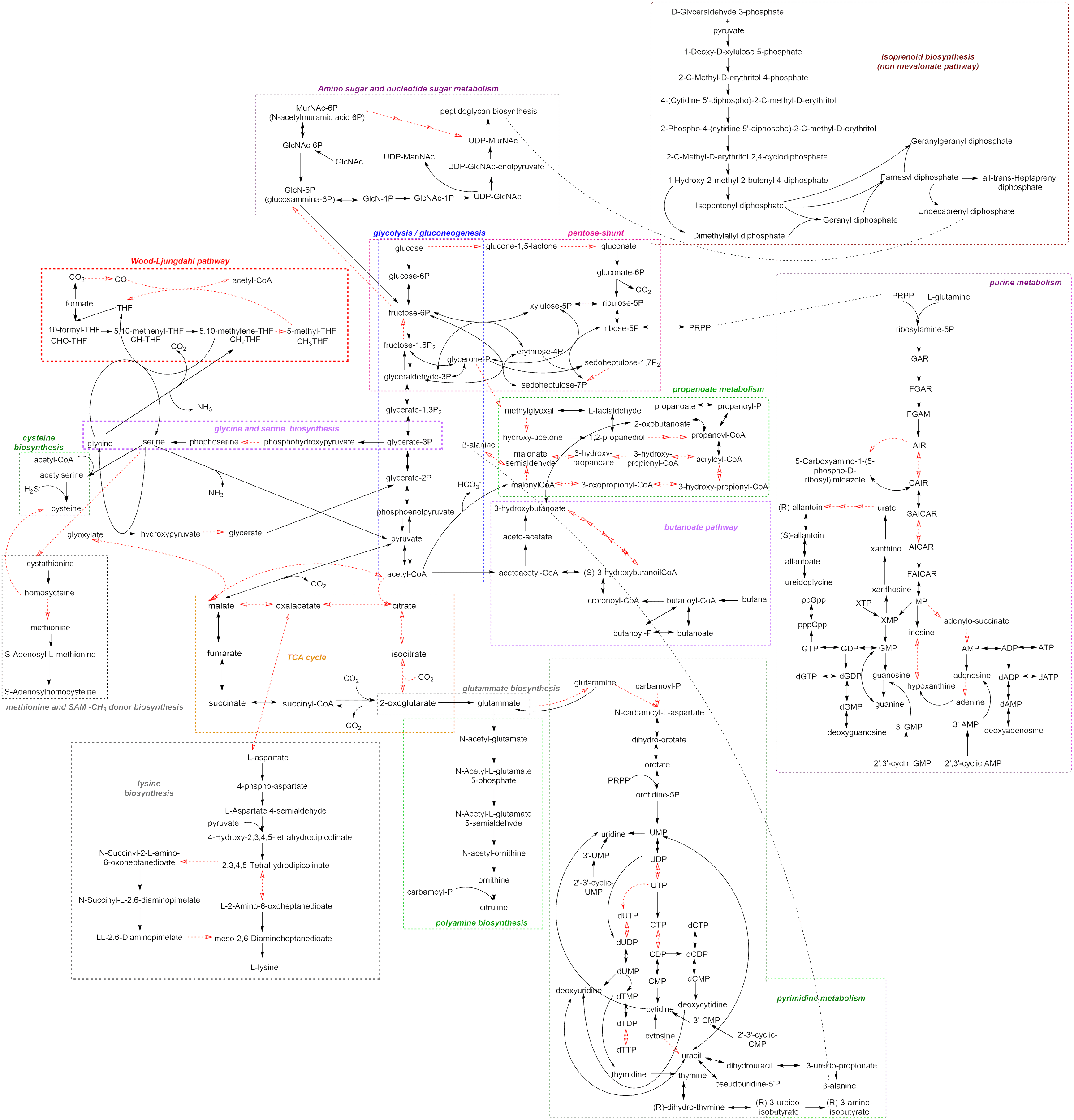
**

**Figure S8. Metabolic reconstruction of Firmicutes sp. GSSM966.**


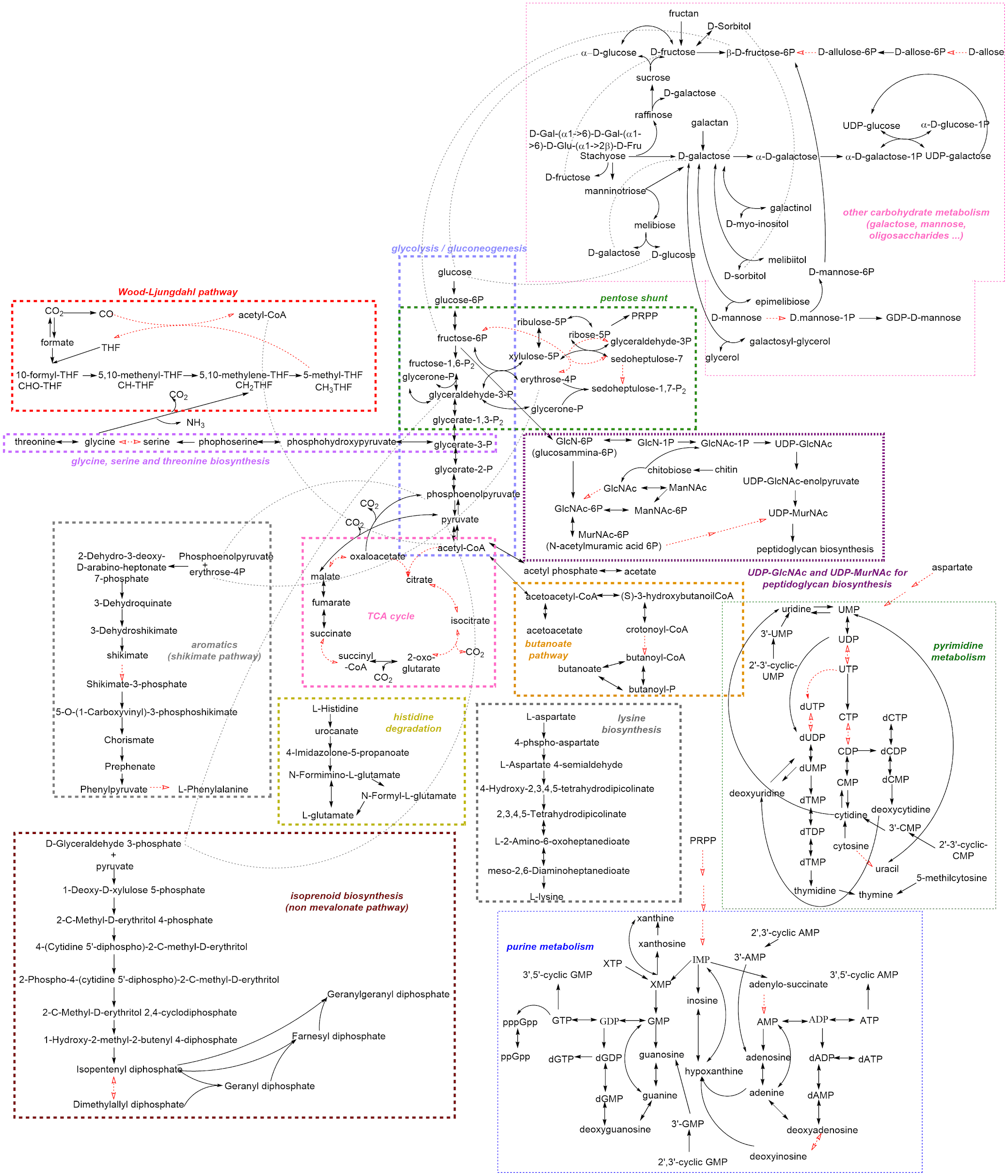


**Figure S9. Metabolic reconstruction of Limnochordia sp. GSSM975.**

**
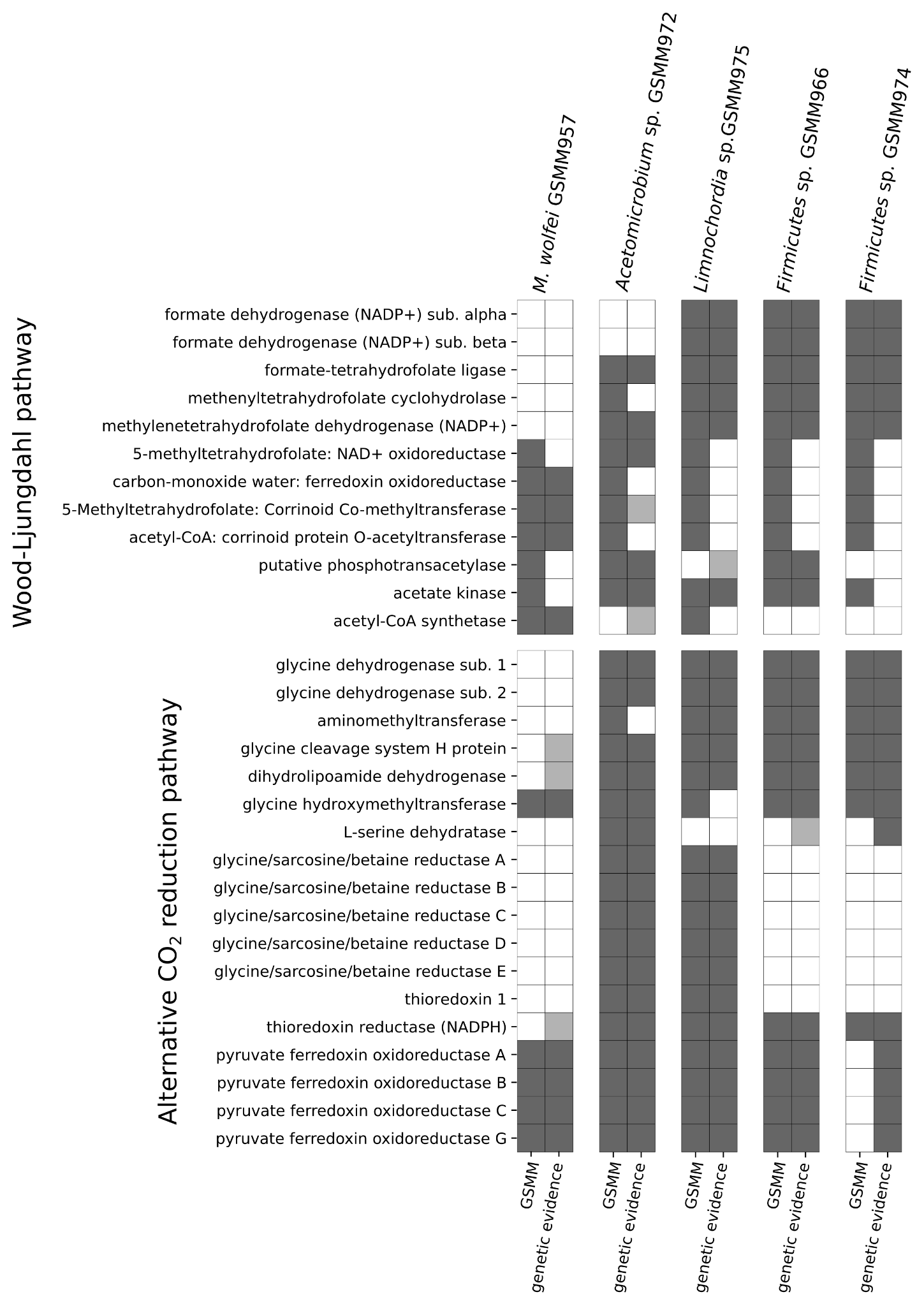
**

**Figure S10. Reactions and corresponding genetic evidence of CO_2_ reduction pathways included in the GSMM.** Summary of reactions belonging to the Wood-Ljungdahl pathway (above) and to the Reductive Glycine Pathway and the Glycine Synthase-Reductase Pathway (below) found to be present/absent in GSMMs. The left section of each heatmaps highlights the reactions included in the GSMM, whereas the right part reports the corresponding genetic evidence in MAGs. Different shades of gray indicate the quality of the result of the homology search as defined by gapseq: “good blast” in dark gray, “bad blast” in light gray and “no blast” in white.

**
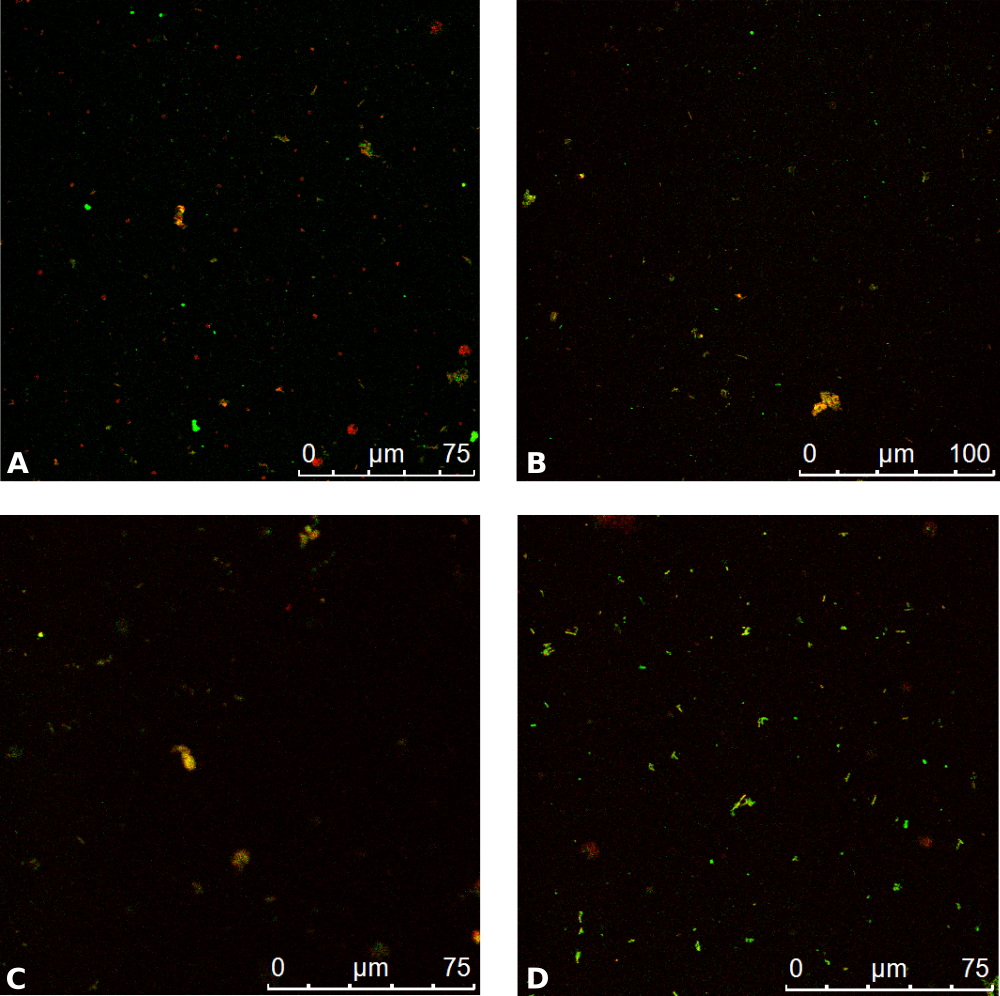
**

**Figure S11. FISH image of the microbial community.** M. wolfeii GSMM957 is labeled with red probes and Limnochordia sp. GSMM975 with green probes.

#### **Datasets S1 to S7**

**Supplementary material. Includes supplementary Materials and methods, Table S1 to S2, Figures S1 to S10.** Supplementary Materials and Methods includes details on genome assembly to obtain maps of the two species of interest and metabolic network reconstruction. Table S1 and S2 includes details on biochemical data of reactor performance. Figure S1 to S10 includes genome maps, metabolic reconstruction and FBA results of the species of interest.

**Dataset S1. Genes responsible for EPS biosynthesis or involved in biofilm formation according to Model Seed categories used for targeted investigation through Hidden Markov Models**. List of KEGG code, reaction and name of genes searched, combined with the number of copies found in each genome. “*” refers to results retrieved by EggNOG annotation alone, “^” refer to results obtained through HMM search and no annotation refers to results retrieved by RAST.

**Dataset S2. Metagenomes clusters used to summarize the abundance profile of MAGs and experiment related unique identifiers of raw reads.** The corresponding reference is associated with each experiment used for coverage profile calculation.

**Dataset S3. KEGG modules completeness level of MAGs.** Integrated results of eggNOG and Diamond annotations.

**Dataset S4. Genome-scale metabolic models reconstructed for the five most abundant species and their quality assessment.** Models are available in SBML format and the reports generated with MEMOTE in HTML format at 10.6084/m9.figshare.16692028.

**Dataset S5. Basic Anaerobic Medium composition used for the community model simulation.** “Compounds” are the unique ModelSEED identifier, “Name” the corresponding human readable metabolites and “MaxFlux” the uptake upper bound allowed form the feedstock.

**Dataset S6. MAG statistics and phylogenetic classification.** General information regarding the recovered 59 MAGs.

**Dataset S7. Operons predicted by Operon Mapper in MAGs.** Results are available at 10.6084/m9.figshare.16691878.

**Dataset S8. Illumina filtered reads and raw nanopore reads quality assessment.** Results obtained with FastQC (v.0.11.9) and NanoPlot (v.1.32.1) are available at 10.6084/m9.figshare.19355303.

#### **References**

1. Rutherford K, Parkhill J, Crook J, Horsnell T, Rice P, Rajandream M-A, et al. Artemis: sequence visualization and annotation. Bioinformatics. 2000;16:944–5.

2. Bassani I, Kougias PG, Treu L, Porté H, Campanaro S, Angelidaki I. Optimization of hydrogen dispersion in thermophilic up-flow reactors for ex situ biogas upgrading. Bioresour Technol. 2017;234:310–9.
