# Supplementary material for: Integrating metagenomic binning with flux balance analysis to unravel syntrophies in anaerobic CO2 methanation
Source: Microbiome. 2022 Aug 3;10:117. doi: 10.1186/s40168-022-01311-1 (PMC9347119; doi:10.1186/s40168-022-01311-1)
Supplement: Supplementary file 10 — Additional file 9: Dataset S8. Illumina filtered reads and raw nanopore reads quality assessment. Results obtained with FastQC (v.0.11.9) and NanoPlot (v.1.32.1) are available at 10.6084/m9.figshare.19355303. [file 40168_2022_1311_MOESM9_ESM.zip › NanoPlot-report.html]

NanoPlot Report

**Summary Statistics**

**Plots**

Histogram of read lengths

Histogram of read lengths after log transformation

Weighted Histogram of read lengths

Weighted Histogram of read lengths after log transformation

Dynamic histogram of Read length

Yield by length

Read lengths vs Average read quality plot using hexagonal bins

Read lengths vs Average read quality plot using dots

# NanoPlot report

## Summary statistics

| feature |  |
| --- | --- |
| General summary |  |
| Mean read length | 4,806.6 |
| Mean read quality | 8.9 |
| Median read length | 1,563.0 |
| Median read quality | 8.9 |
| Number of reads | 591,997.0 |
| Read length N50 | 13,708.0 |
| STDEV read length | 7,182.0 |
| Total bases | 2,845,482,642.0 |
| Number, percentage and megabases of reads above quality cutoffs |  |
| >Q5 | 591997 (100.0%) 2845.5Mb |
| >Q7 | 591997 (100.0%) 2845.5Mb |
| >Q10 | 105125 (17.8%) 841.6Mb |
| >Q12 | 406 (0.1%) 1.4Mb |
| >Q15 | 0 (0.0%) 0.0Mb |
| Top 5 highest mean basecall quality scores and their read lengths |  |
| 1 | 13.7 (460) |
| 2 | 13.6 (681) |
| 3 | 13.4 (461) |
| 4 | 13.2 (808) |
| 5 | 13.2 (271) |
| Top 5 longest reads and their mean basecall quality score |  |
| 1 | 92412 (7.2) |
| 2 | 81958 (8.0) |
| 3 | 79432 (10.4) |
| 4 | 78922 (10.9) |
| 5 | 77975 (10.2) |

## Plots

### Histogram of read lengths

  
  
  
  

### Histogram of read lengths after log transformation

  
  
  
  

### Weighted Histogram of read lengths

  
  
  
  

### Weighted Histogram of read lengths after log transformation

  
  
  
  

### Dynamic histogram of Read length

  
  
  
  

### Yield by length

  
  
  
  

### Read lengths vs Average read quality plot using hexagonal bins

  
  
  
  

### Read lengths vs Average read quality plot using dots
